# SARS-CoV-2 infection induces a pro-inflammatory cytokine response through cGAS-STING and NF-κB

**DOI:** 10.1038/s42003-021-02983-5

**Published:** 2022-01-12

**Authors:** Christopher J. Neufeldt, Berati Cerikan, Mirko Cortese, Jamie Frankish, Ji-Young Lee, Agnieszka Plociennikowska, Florian Heigwer, Vibhu Prasad, Sebastian Joecks, Sandy S. Burkart, David Y. Zander, Baskaran Subramanian, Rayomand Gimi, Seetharamaiyer Padmanabhan, Radhakrishnan Iyer, Mathieu Gendarme, Bachir El Debs, Niels Halama, Uta Merle, Michael Boutros, Marco Binder, Ralf Bartenschlager

**Affiliations:** 1 Department of Infectious Diseases, Molecular Virology, Heidelberg University, Heidelberg, Germany; 2 BioMed X Institute, BioMed X GmbH, Heidelberg, Germany; 3 Division Virus-Associated Carcinogenesis, German Cancer Research Center, Heidelberg, Germany; 4 Division of Signaling and Functional Genomics, German Cancer Research Center, and Department of Cell and Molecular Biology, Heidelberg University, Medical Faculty Mannheim, Mannheim, Germany; 5 Research Group “Dynamics of Early Viral Infection and the Innate Antiviral Response”, Division Virus-Associated Carcinogenesis, German Cancer Research Center, Heidelberg, Germany; 6 Spring Bank Pharmaceuticals, Inc., 35 Corporate Drive, Hopkinton, MA 01748 USA; 7 Division of Translational Immunotherapy, German Cancer Research Center (DKFZ), Heidelberg, Germany; 8 Department of Internal Medicine IV, University Hospital Heidelberg, Heidelberg, Germany; 9 German Center for Infection Research, Heidelberg partner site, Heidelberg, Germany

**Keywords:** SARS-CoV-2, Viral pathogenesis

## Abstract

SARS-CoV-2 is a novel virus that has rapidly spread, causing a global pandemic. In the majority of infected patients, SARS-CoV-2 leads to mild disease; however, in a significant proportion of infections, individuals develop severe symptoms that can lead to long-lasting lung damage or death. These severe cases are often associated with high levels of pro-inflammatory cytokines and low antiviral responses, which can cause systemic complications. Here, we have evaluated transcriptional and cytokine secretion profiles and detected a distinct upregulation of inflammatory cytokines in infected cell cultures and samples taken from infected patients. Building on these observations, we found a specific activation of NF-κB and a block of IRF3 nuclear translocation in SARS-CoV-2 infected cells. This NF-κB response was mediated by cGAS-STING activation and could be attenuated through several STING-targeting drugs. Our results show that SARS-CoV-2 directs a cGAS-STING mediated, NF-κB-driven inflammatory immune response in human epithelial cells that likely contributes to inflammatory responses seen in patients and could be therapeutically targeted to suppress severe disease symptoms.

## Abstract (web summary)

Neufeldt et al. evaluate transcriptional and cytokine secretion profiles of cells and patient sera following SARS-CoV-2 infection and detect distinct upregulation of inflammatory cytokines. They also demonstrate that this upregulation is mediated by cGAS-STING and NF-κB signalling, which could provide a potential avenue for the development of future therapies against SARS-CoV-2.

## Introduction

In late 2019 SARS-CoV-2 emerged as a highly infectious coronavirus that causes respiratory disease in humans, termed COVID-19. Since the initial identification, SARS-CoV-2 has spread around the world leading the World Health Organization to declare a pandemic. The SARS-CoV-2 infection causes respiratory symptoms that range from mild to severe and can result in lasting lung damage or death in a significant number of cases^1^. One of the hallmarks of severe COVID-19 is the low levels of type I interferons (IFNs) and overproduction of inflammatory cytokines or chemokines such as IL-6 and TNF^2–5^. This unbalanced immune response fails to limit virus spread and can cause severe systemic symptoms^3,6^. Therapies aimed at modulating immune activation to attenuate the detrimental inflammatory response or promote an antiviral cytokine response represent an important avenue for treating patients with severe COVID-19.

During virus infection, the specific immune signals produced from infected cells are important for dictating the recruitment and activation of innate or adaptive immune cells that are required to fight virus infection. For SARS-CoV-2, lung epithelial cells are the primary site of infection and therefore are responsible for initiating immune responses to virus infection. Like all plus-strand RNA viruses, the SARS-CoV-2 replication process within cells requires de novo production of viral RNA species, including single-strand (ss)RNA and double-strand (ds)RNA that can be sensed by cytosolic pattern recognition receptors (PRRs) subsequently activating antiviral pathways^7^. In addition to direct viral sensing, cells have also evolved ways to detect the indirect effects of virus infection, such as nuclear or mitochondrial damage caused by the heavy cellular burden imposed by virus replication. Cytoplasmic DNA sensors including cGAS-STING, IFI16, or AIM2, recognize dsDNA from DNA viruses, but also play an important role in RNA virus infection, either through directly recognizing viral signatures or through sensing of cellular DNA released from mitochondria or nuclei due to cellular stress (reviewed in^8,9^). Moreover, cGAS is an important molecule for regulating basal expression levels of cell-intrinsic immune genes in cells and is, therefore, a central protein in immune responses to virus infection^10^. Substrate recognition by either RNA or DNA sensors leads to signaling cascades that activate two major branches of the innate immune response, the type I/III IFN response and the inflammatory cytokine response (reviewed in ref. ^11^). The type I/III IFN pathways are directly involved in protecting neighboring cells from virus spread and are vital for the immediate cell-intrinsic antiviral response. The inflammatory cytokine response is involved in the recruitment and activation of immune cells.

Plus-strand RNA viruses have evolved numerous ways to limit or block these cellular immune pathways. For SARS-CoV-2 infection, initial transcriptional analyses of infected cells have generated ambiguous results on the induction of type I/III IFNs and the subsequent expression of IFN stimulated genes (ISGs). On the one hand, it was shown that SARS-CoV-2 triggers only an attenuated immune response in both immortalized and primary cell lines, suggesting a block in PRR signaling pathways^12,13^. On the other hand, several studies argue for strong induction of IFN responses in both lung and intestinal infection models^14,15^. In systems where infection causes high levels of IFN activation, immune sensing of viral RNA is through the cytosolic RNA sensor MDA5, leading to an activation of the signaling molecules mitochondrial antiviral-signaling (MAVS) and TANK-binding kinase 1 (TBK1)^16–18^. However, IFN production has only been observed at late time-points after SARS-CoV-2 infection, suggesting that activation of bystander cells rather than the initial infection leads to IFN activation. Other studies have indicated that virus-encoded proteins actively target and antagonize multiple steps of the immune activation pathway leading to a robust block in the IRF3 mediated IFN response^19^. This includes limiting activation of the key signaling proteins or kinases such as MAVS or TBK1, or through perturbing the function of IRF3, which is a key transcription factor in activating IFN responses^20–26^. SARS-CoV-2 infection has also been shown to inhibit downstream IFN signaling by attenuating JAK/STAT signaling^27^. The range of varying observations in cell-intrinsic immune response activation or repression leaves several open questions as to how SARS-CoV-2 infection modulates immune responses in infected epithelial cells and how these initial responses can lead to either viral clearance or severe disease.

Here, we report the transcriptomic profiles derived from SARS-CoV-2 infected human lung cells showing a specific bias towards an NF-κB mediated inflammatory response and a restriction in the TBK1 specific IRF3 activation and subsequent IFN response. Consistently, cytokine profiles from both severe COVID-19 patients and SARS-CoV-2 infected lung epithelial cells were enriched for pro-inflammatory cytokines, specifically IL-6, and lacked type I/III IFNs. We also demonstrate that SARS-CoV-2 infection leads specifically to NF-κB, but not IRF3 nuclear localization, and that poly(I:C)-induced pathway activation is attenuated in infected cells. Finally, we show that the cGAS-STING pathway is activated by SARS-CoV-2 infection, leading to a specific NF-κB response and that inflammatory cytokine upregulation can be mitigated by several drugs that inhibit STING. These results provide insight into how innate immune responses are modulated by SARS-CoV-2 in epithelial cells likely contributing to the initiation of the hyper-inflammatory responses observed in severe COVID-19 cases.

## Results

### Kinetics of SARS-CoV-2 infection in lung epithelial cells

SARS-CoV-2 predominantly targets airway and lung tissue in infected individuals. In order to determine the effects of SARS-CoV-2 on human lung epithelial cells, Calu-3 cells were infected with SARS-CoV-2 and the host transcriptional profiles were determined over the time course of infection. Following infection, we observed an increase in intracellular viral RNA starting at 4 h post infection, which continued to increase up to 24 h post infection (Supplementary Fig. 1a). Increased extracellular viral RNA was observed starting at 6 h post infection, which was paralleled by the release of infectious virus (Supplementary Fig. 1a, b).

**Fig. 1:**
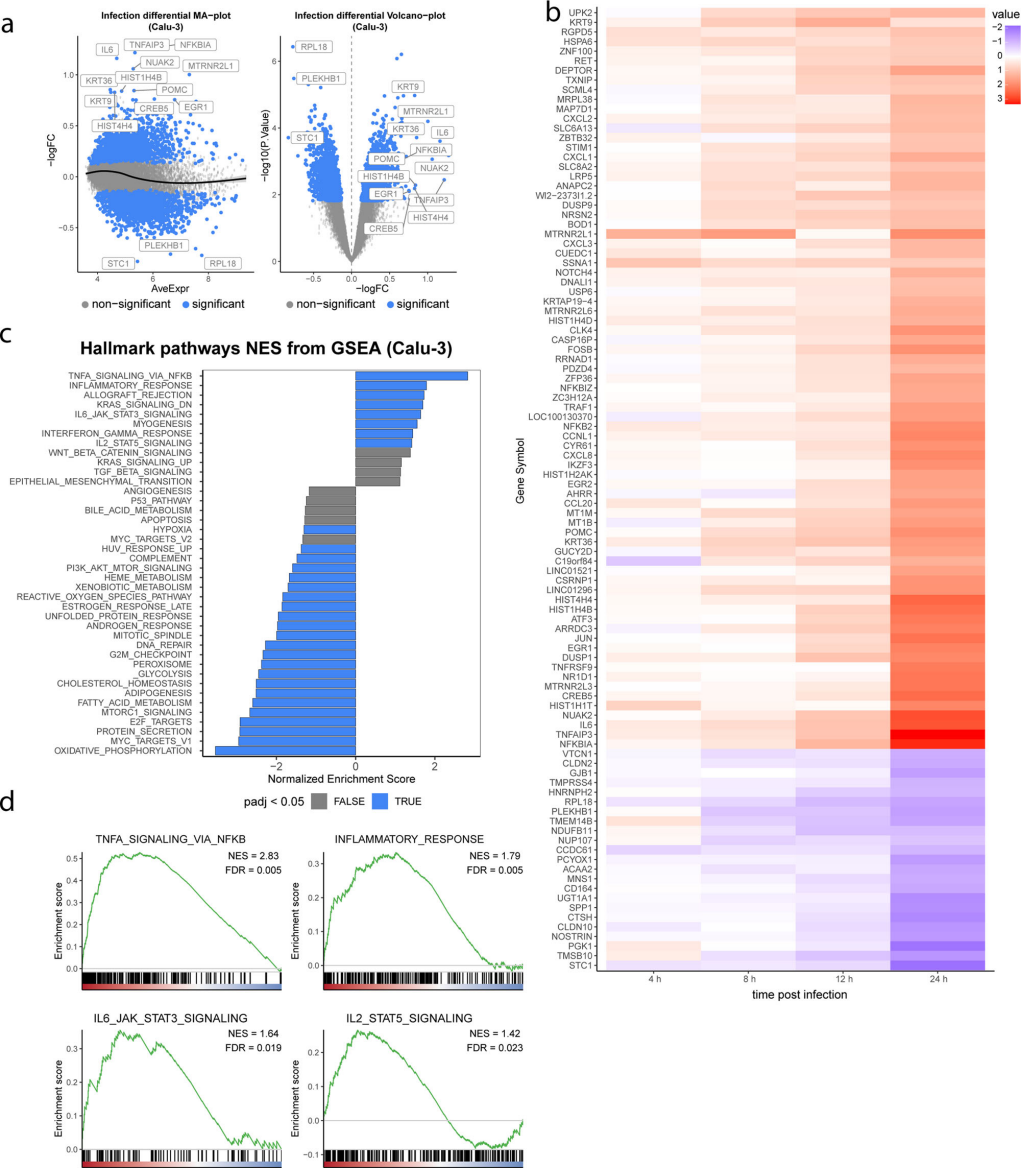
Transcriptional changes induced upon SARS-CoV-2 infection over time. Calu-3 cells were infected with SARS-CoV-2. At the indicated time-points after infection, total RNA was harvested, and mRNA transcript levels were determined by Illumina microarray. Probe intensities were quantile normalized using probe-wise normalization. Normalized probe intensities were averaged for each gene and log-transformed. Differential expression was then calculated using a standard R/limma workflow for microarray analysis. **a** MA-plots and Volcano-plots of transcriptional changes in Calu-3 cells highlighting differentially expressed genes. Blue dots represent significant changes as determined by R/limma with a Benjamini–Hochberg adjusted *p* value smaller than 0.1. Left plot (MA-plot) shows log2 fold change on the y-axis vs mean normalized expression on the x-axis. Right plot (volcano-plot) shows the significant hits considering both infection and changes over time (x-axis = log2 fold change; y-axis = −log10 *p* value; top 15 significant genes marked). **b** Heat map of log scaled relative expression of enriched genes in Calu-3 cells. **c** Gene set enrichment analysis employing the MSigDB collection of Hallmark pathways for Calu-3 cells. Top 40 enriched pathways for up- or downregulated genes are shown, ranked by their normalized enrichment score. Color indicates significance (blue = adjusted *p* value < 0.05). **d** Barcode plots for pathways showing a significant number of upregulated genes following SARS-CoV-2 infection in Calu-3 cells. NES = normalized area under the curve. FDR = Benjamini–Hochberg adjusted *p* value of enrichment (false discovery rate). For transcriptome analysis, *n* = 2 biological replicates.

To determine the effects of SARS-CoV-2 infection on the host transcriptional profile, total RNA was isolated from infected cells and analyzed by microarray (Fig. 1 and Supplementary Fig. 1). For these studies, we took into account that the SARS-CoV-2 replication cycle in cell culture is 6–16 h, followed by the death of infected cells^28^. Therefore, to evaluate the direct effects of virus infection and avoid the analysis of secondary infection events or cell death effects, we did not extend our analysis beyond 24 h post infection. Analysis of significantly differentially expressed genes showed substantial transcriptomic changes in Calu-3 cells with a total of 3215 differentially expressed genes (FDR <10%, Fig. 1a). Principal component analyses (PCA) showed significant effects of SARS-CoV-2 infection on Calu-3 cells, especially at 24 h post infection (Supplementary Fig. 1a–c). We did not observe an overall decrease in total mRNA quality or large differences in probe intensity, thus showing no indication that SARS-CoV-2 infection causes a general transcriptional shutdown. Gene set enrichment analysis of the transcriptional changes using curated “Hallmark” pathways showed a strong upregulation of inflammatory responses, with gene sets from NF-κB and IL-6-STAT3 pathways showing a high degree of enrichment (Fig. 1b–d)^29^. Interestingly, transcripts involved in the type I/III IFN pathways showed little change over the course of infection. These results suggest that in human lung cells, the response to SARS-CoV-2 infection is dominated by pro-inflammatory, NF-κB-driven pathways, with little contribution of the antiviral IFN system.

To determine if transcriptional changes induced by SARS-CoV-2 infection were conserved in other cell lines, the transcriptional profiles from Calu-3 cells were compared to profiles from infected A549 cells that express the ACE2 receptor (A549-ACE2). Although the levels of viral RNA, production of infectious virus, and virus spread were higher in Calu-3 cells compared to A549-ACE2 cells, we observed a high degree of overlap between top significantly upregulated and downregulated gene sets from both cell lines (Supplementary Fig. 1d–h). These results indicated that the activation of pro-inflammatory cellular pathways in SARS-CoV-2 infection is conserved between different cell lines.

### Pro-inflammatory activation in SARS-CoV-2 infected lung epithelial cells parallels patient responses

**Fig. 2:**
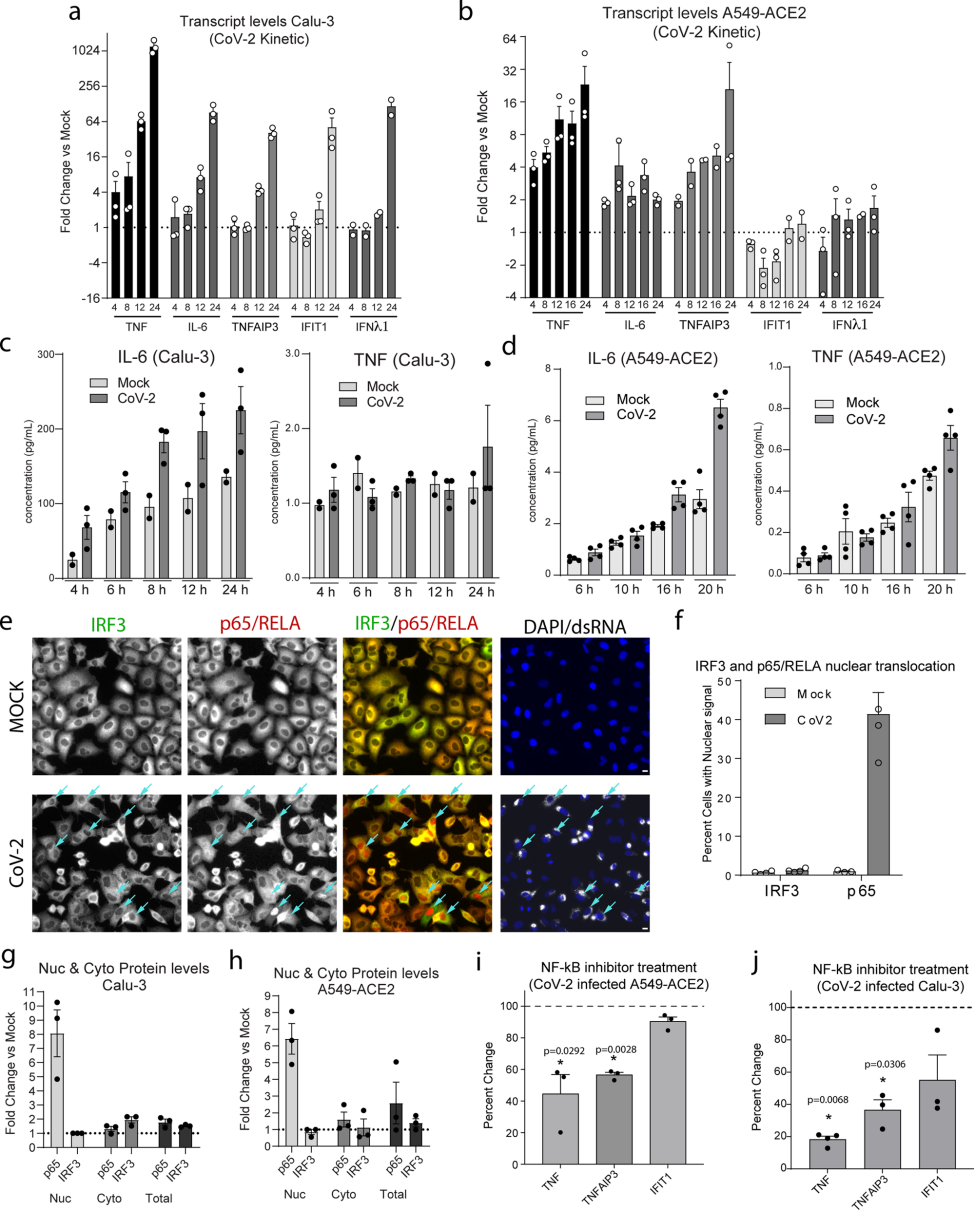
SARS-CoV-2 specifically activates the NF-κB pathway but not IFN/ISGs. **a–d** Calu-3 or A549-ACE2 cells were infected with SARS-CoV-2. At the indicated time-points post infection, total cellular RNA and cell culture supernatants were harvested from mock cells and infected cells. **a**, **b** Levels for the indicated mRNA transcripts were determined at each time-point post infection of Calu-3 cells (panel a) or A549-ACE2 cells (panel b) by RT-qPCR. Graphs show the fold change in the indicated mRNA transcript levels for the SARS-CoV-2 samples compared to mock samples from the same time-point. The y-axis scale is displayed in log2 increments. **c**, **d** Supernatant samples from infected A549-ACE2 or Calu-3 cells were treated with beta-propiolactone (for biosafety reasons) and the cytokine profiles were determined by using the MDS platform (**c**) or by flow cytometry using the LGENDplex antiviral response panel (**d**). Graphs show the mean concentration for each cytokine (pg/mL) for mock and infected cells. **e**, **f** A549-ACE2 cells were infected with SARS-CoV-2 for 16 h. **e** Cells were fixed and stained with antibodies specific for IRF3 (green), p65/RELA (red), and dsRNA (gray). Turquoise arrows point to cells showing p65/RELA nuclear accumulation. Scale bars, 10 μm. **f** Graph shows the mean nuclear accumulation of IRF3 and p65/RELA for images from cells treated as in panel (**e**). **g**, **h** Calu-3 or A549-ACE-2 cells were infected with SARS-CoV-2 for 16 h followed by subcellular fractionation into Nuclear (Nuc), Cytosolic (Cyto), and whole-cell lysate (Total) fractions using centrifugation. Protein levels for p65/RELA and IRF3 were determined by western blotting. Graphs show the mean fold change in protein levels in SARS-CoV-2 infected cells compared to mock. Protein levels were corrected for GAPDH. Corresponding western blots are shown in Supplemental Fig. S3c, d. **i**, **j** A549-ACE2 or Calu-3 cells were infected with SARS-CoV-2 and, 1 h later, cells were treated with the NF-κB inhibitor (BI-605906) or DMSO only. The mRNA transcript levels for the given genes were determined by RT-qPCR. Mock indicates fold change between transcript levels in uninfected cells (BI-605906 mock vs DMSO mock) and CoV-2 indicates fold change in transcript levels between infected cells (BI-605906 CoV-2 vs DMSO CoV-2). The graphs show the mean fold change for three independent experiments corrected for HPRT. For all graphs, statistical significance was determined using the student’s *t*-test. * represents statistical significance and exact *p* values are provided. For all panels, *n* ≥ 3 biological replicates.

Transcriptional activation of NF-κB and inflammatory cytokine pathways in cultured cells infected with SARS-CoV-2 indicates that infected epithelial cells might contribute directly to the initiation of cytokine responses observed in severe COVID-19^2^. To confirm our bulk transcriptome analysis, we evaluated the mRNA transcript levels of representative pro-inflammatory or IFN pathway genes following SARS-CoV-2 infection in both Calu-3 and A549-ACE2 cells. In agreement with our transcriptome analysis, we observed significant mRNA increases of pro-inflammatory cytokine genes including *TNF*, *IL-6*, and *TNFAIP3* starting at 4 h post infection, while increases in IFN pathway genes were only observed at 24 h and only in Calu-3 cells (Fig. 2a, b). To test if gene upregulation resulted in the secretion of cytokines that could contribute to the spread of pro-inflammatory signals, we compared the levels of secreted cytokines from infected Calu-3 or A549-ACE2 cells to cytokine levels in serum taken from infected patients with severe COVID-19 (Fig. 2c, d and Supplementary Fig. 2a, c). Consistent with previous reports, infected patients had elevated levels of inflammatory cytokines, notably IL-6, IL-8, and IP-10 (Supplementary Fig. 2c)^2^. An upregulation in *IL-6* transcription and protein secretion was also found in A549-ACE2 cells and, more pronounced, in Calu-3 cells infected with SARS-CoV-2 (Fig. 2c, d and Supplementary Fig. 2a, b). Importantly, the levels of IL-6 protein secretion are consistent with previously published levels released from activated epithelial cells^30–32^. In both cell lines, although significant increases in *TNF* mRNA transcripts were seen following infection, only modest increases in TNF cytokine levels were observed (Fig. 2a, b compared to Fig. 2c, d). This could be due to defects in TNF processing or secretion into the supernatant in these cell lines or limitation in the sensitivity of the assay.

Secreted IL-6 levels were elevated starting at 4 h after SARS-CoV-2 infection, indicating that later activation of pro-inflammatory cytokines might be the result of a paracrine response. To test this assumption, the level of pro-inflammatory gene activation was measured in the presence of IL-6 or TNF neutralizing antibodies following SARS-CoV-2 infection of Calu-3 or A549-ACE2 cells (Supplementary Fig. 2d–f). Importantly, the level of IL-6 or TNF neutralizing antibodies used was sufficient to decrease the cytokine-mediated response by >50% in uninfected cells (Supplementary Fig. 2d). In SARS-CoV-2 infected cells, neither IL-6 nor TNF neutralizing antibodies significantly altered pro-inflammatory gene levels (Supplementary Fig. 2e, f), indicating that activation of this response is not due to paracrine action of these cytokines.

Paralleling reports from other groups, we observed elevation of secreted cytokine and transcript levels of type III IFNs and the ISG *IFIT1* in infected Calu-3 cells, but not in A549-ACE2 cells, at 24 h post infection^16,17^. To further characterize this late induction of the IFN response, we evaluated cytokine transcript and viral RNA levels over a 72 h time course of infection. In Calu-3 cells, we observed an increase in *IFNβ* and *IFIT1* transcript levels, starting at 24 h and continuing to increase up to 72 h while viral RNA levels peaked at 24 h (Supplementary Fig. 2g). In A549-ACE2 cells, we also observed a slight increase in *IFNβ* transcript levels at 24 h and *IFIT1* transcript levels at 48 h post infection (Supplementary Fig. 2h). However, significant activation of the *IFIT1* promoter was not observed using a previously described GFP reporter system (Supplementary Fig. 2i, j)^33^. Notably, for both cell lines, a robust early increase in *TNF* and *TNFAIP3* was observed that paralleled viral RNA levels. These results indicate that the late induction of IFN in Calu-3 cells arises after the peak of RNA replication and is therefore likely not directly due to the initial infection. Rather, this induction is likely a result of virus spread into pre-activated bystander cells.

To determine if the pro-inflammatory response induced by SARS-CoV-2 infection parallels other plus-strand RNA virus infections, we compared the induction of pro-inflammatory or IFN genes between SARS-CoV-2 and Zika virus infected cells (Supplementary Fig. 3a, b). Following Zika virus infection in both A549-ACE2 and Calu-3 cells, we observed robust activation of the IFNβ gene compared to the limited activation in SARS-CoV-2 infected cells, even though viral RNA levels were higher for SARS-CoV-2 infection. Of note, we did not observe changes in *TNF* mRNA levels following incubation of non-infectable wild type A549 cells with SARS-CoV-2, confirming that the induction of *TNF* expression in A549-ACE2 cells results from virus infection and is not a paracrine response from virus stock production in VERO cells.

Together, these results corroborate published data showing that, in cases with high viral load, SARS-CoV-2 infection preferentially induces pro-inflammatory cytokine production with little activation of the antiviral responses^12,17,34^. Additionally, these data indicate that infected epithelial cells secrete cytokines that could contribute to the initiation of tissue-level inflammation, a response that is likely propagated by innate immune cells.

### SARS-CoV-2 infection specifically activates NF-κB but not IRF3

The high levels of inflammatory gene activation and the poor activation of IFNs and ISGs in response to SARS-CoV-2 infection led us to investigate which transcription factors of the cell-intrinsic immunity are activated by the virus. In general, sensing of viral infection in epithelial cells by cytosolic innate immune receptors leads to the parallel activation (i.e., phosphorylation) and nuclear accumulation of the two hallmark transcription factors IRF3 and NF-κB. To evaluate the impact of SARS-CoV-2 infection on these pathways, we quantified the nuclear translocation of IRF3 and NF-κB in infected A549-ACE2 cells by using light microscopy (Fig. 2e, f). Consistent with our transcriptomic data showing limited activation of antiviral genes, we observed no significant nuclear accumulation of IRF3 in SARS-CoV-2 infected cells. Conversely, a significant portion of infected cells showed nuclear translocation of NF-κB (Fig. 2e, f). This selective nuclear accumulation of NF-κB but not IRF3 was also observed following subcellular fractionation of SARS-CoV-2 infected cells and western blot analysis of the fractions (Fig. 2g, h and Supplementary Fig. 3c). In line with this, western blotting showed increased levels of phosphorylated NF-κB p65/RELA starting at 12 h post infection, accompanied by a decrease in IκB levels (Supplementary Fig. 3d–f). Consistent with the imaging data, we did not detect an increase in IRF3 phosphorylation following infection (Supplementary Fig. 3g).

To further confirm the role of NF-κB in SARS-CoV-2 induced inflammation, we evaluated the effects of a specific IKK inhibitor (BI-605906) on the transcriptional profile of genes activated by NF-κB following infection^35^. Treatment of infected cells with BI-605906 limited both *TNF* and *TNFAIP3* transcriptional activation following infection (Fig. 2i, j). These results suggest that in SARS-CoV-2 infection, NF-κB is selectively activated while IRF3 activation appears to be circumvented or suppressed.

### Inflammatory response to SARS-CoV-2 infection is mediated by cGAS-STING and not RNA sensors

To determine the source of the SARS-CoV-2 induced inflammatory response or downstream immune activation, we evaluated the effects of innate immune receptor knockout or overexpression. We first looked at RNA receptors that have previously been described to recognize viral RNAs including RIG-I, MDA5, and TLR3 as well as IFN receptors. RIG-I/MDA5 double knockout, TLR3 overexpression, or IFN receptor (IFNAR, IFNGR, IFNLR) triple knockout A549-ACE2 cells were infected with SARS-CoV-2 and the transcriptional upregulation of *IFIT1* (IRF3 target) and *TNF* (NF-κB target^36–38^) transcript levels were used as readouts for pathway activation. No significant changes in *IFIT1* mRNA, *TNF* mRNA levels or viral RNA levels were observed in any of the cell lines compared to control cells (Supplementary Fig. 4a, b). Furthermore, we found that in SARS-CoV-2 infected cells, transfection with the dsRNA mimic poly(I:C) had a significantly lower effect than in uninfected cells (Supplementary Fig. 4c, d). This reduction in poly(I:C) immune induction was specifically limited to infected cells within the population (Supplementary Fig. 4e, f). Together, these data indicate that in the cell culture systems used here, SARS-CoV-2 infection robustly blocks the activation of immune pathways through RNA-specific PRRs. This also suggests that recognition of viral RNA via cellular RNA sensors is not involved in NF-κB activation in SARS-CoV-2 infected cells.

**Fig. 3:**
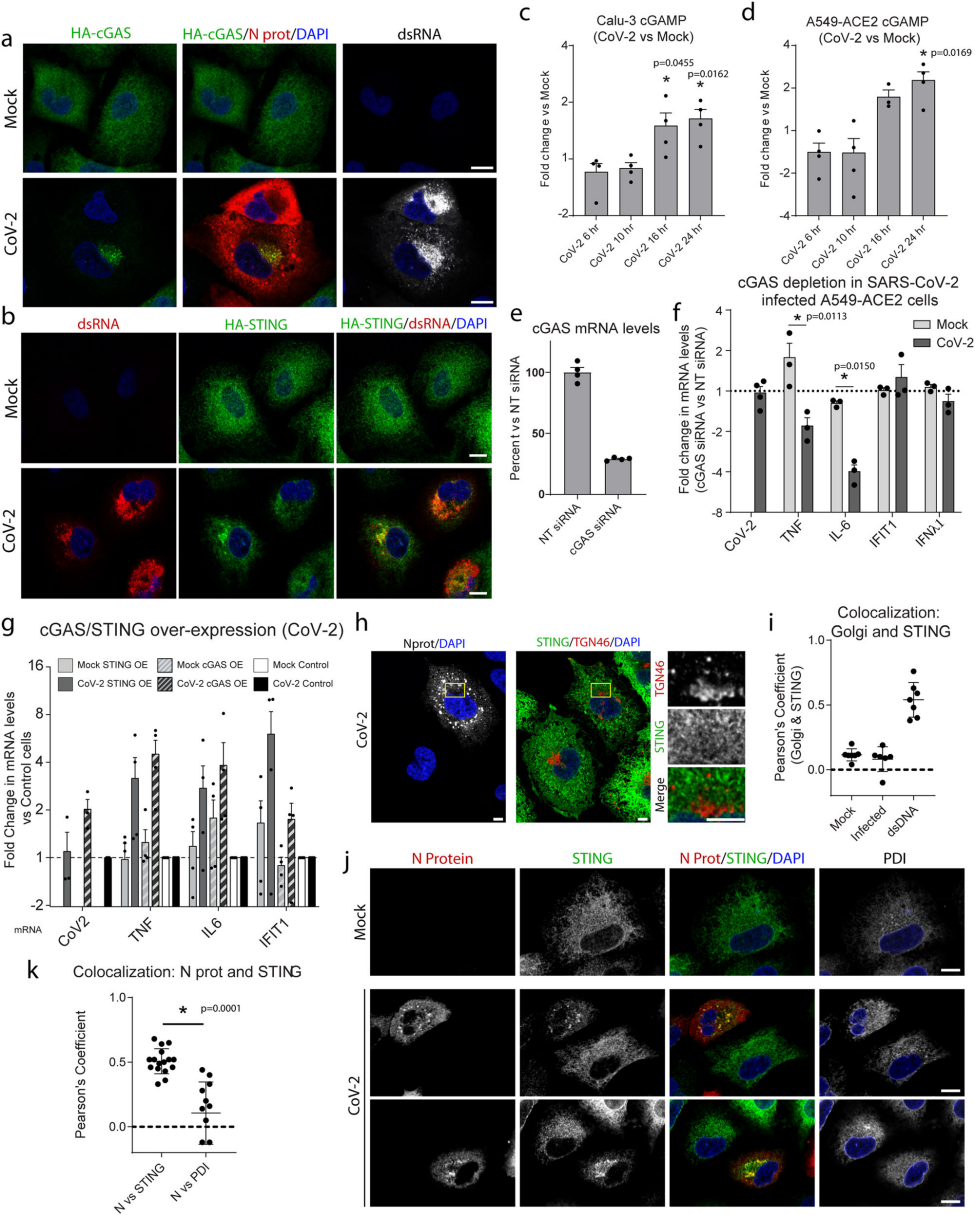
cGAS-STING activation mediates the NF-κB response in SARS-CoV-2 infected cells. **a**, **b** A549-ACE2 cells stably expressing HA-cGAS (**a**) or HA-STING (**b**) were infected with SARS-CoV-2 for 16 h followed by fixation and staining with the indicated antibodies. Cells were analyzed by confocal microscopy. Scale bars 10 μm. **c**, **d** Calu-3 or A549-ACE2 cells were infected with SARS-CoV-2. Whole-cell lysates from infected and uninfected cells were harvested at the indicated time-points. Intracellular cGAMP levels were evaluated by ELISA and corrected for total cellular protein levels. Graphs show the average fold change in cGAMP levels between infected and uninfected cells at the same time-point after infection. The y-axis scale is displayed in log2 increments. **e**, **f** A549-ACE2 cells were transfected with siRNAs directed against cGAS or non-targeting (NT) control siRNAs. After 2 days, cells were infected with SARS-CoV-2 for 16 h followed by isolation of cellular RNA. **e** cGAS mRNA levels were determined by RT-qPCR. The graph shows the cGAS mRNA levels as a percentage of the control cells. **f** mRNA transcript levels for the given genes were determined by RT-qPCR. Mock indicates fold change between transcript levels from uninfected cells (cGAS siRNA mock vs NT siRNA mock) and CoV-2 indicates fold change in transcript levels between infected cells (cGAS siRNA infected vs NT siRNA infected). The y-axis scale is displayed in log2 increments. **g** A549-ACE2 cells stably expressing STING, cGAS, or a control plasmid were infected with SARS-CoV-2 for 16 h. The levels of viral RNA or the indicated host mRNA transcripts were determined by RT-qPCR. Mock indicates fold change between transcript levels in uninfected cells (overexpression cell mock vs control cell mock) and CoV-2 indicates fold change in transcript levels between infected cells (overexpression cell infected vs control cell infected). The y-axis scale is displayed in log2 increments. **h**–**k** A549-ACE2 cells expressing HA-STING were infected with SARS-CoV-2 for 16 h followed by fixation. **h** Cells stained with the indicated antibodies were analyzed by confocal microscopy. Scale bars 10 μm. **i** Pearson’s correlation coefficient for fluorescence signal pertaining to STING and TGN46 Golgi signal in uninfected cells, SARS-CoV-2 infected cells, or uninfected cells transfected with herring testes DNA (hDNA). *N* > 20 cells. **j** Cells stained with the indicated antibodies were analyzed by confocal microscopy. Scale bars 10 μm. **k** Pearson’s correlation coefficient for fluorescence signal pertaining to SARS-CoV-2 N protein compared to either STING fluorescence signal or PDI fluorescence signal. *N* > 20 cells. For all panels, *n* ≥ 3 biological replicates.

The induction of the cGAS-STING-signaling axis leading to the activation of NF-κB and IRF3 has been reported for several RNA virus infections, most likely through cellular stress responses to viral infection^9,10,39–42^. To determine whether the cGAS-STING pathway is triggered in SARS-CoV-2 infected cells, we first evaluated changes in the localization of cGAS or STING in infected cells. Indeed, both cGAS and STING were observed to re-localize to perinuclear clusters in infected cells, indicative of activation (Fig. 3a, b). Similar re-localization of Sec-61β was not observed, indicating that cGAS and STING clustering is not an effect of SARS-CoV-2-mediated ER reorganization (Supplementary Fig. 4g). To confirm the activation of cGAS, we evaluated the cellular levels of cyclic GMP-AMP (cGAMP), which is produced by cGAS following recognition of dsDNA^43–45^. Increases in cellular cGAMP levels, consistent with physiologic mitochondrial stress activation^46,47^, were observed starting at 16 h after SARS-CoV-2 infection of either A549-ACE2 or Calu-3 cells, further indicating cGAS activation during infection (Fig. 3c, d and Supplementary Fig. 4h, i). Additionally, we observed that, unlike in poly(I:C)-mediated activation of RLRs, SARS-CoV-2 infection did not interfere with the activation of the cGAS-STING pathway by dsDNA transfection (Supplementary Fig. 4j, k).

In order to determine if the cGAS/STING axis is directly involved in the induction of inflammatory cytokines in SARS-CoV-2 infected cells, depletion and overexpression experiments were performed. Since Calu-3 cells were resistant to genetic manipulation, A549-ACE2 cells, which show consistent inflammatory pathway activation, were used to evaluate the effect of depletion or overexpression in SARS-CoV-2 infected cells. A549-ACE2 cells were depleted of cGAS using siRNA followed by infection with SARS-CoV-2. In cGAS knockdown cells, we observed that, although viral RNA levels were unchanged, there was a significant decrease in *TNF* and *IL-6* mRNA transcript levels following infection (Fig. 3e, f). Importantly, we did not observe significant changes in inflammatory gene mRNA transcript levels between uninfected cells transfected with control siRNA or cGAS siRNA (Fig. 3f, mock bars). For overexpression experiments, A549-ACE2 cells stably expressing STING, cGAS, or an empty control plasmid were infected with SARS-CoV-2 (Fig. 3g). Overexpression of either cGAS or STING caused increased inflammatory gene transcriptional activation in infected cells. Notably, even though a slight elevation in *IL-6* and *IFIT1* mRNA transcripts was observed in uninfected cells expressing cGAS or STING, the increases observed in infected cells were higher.

Although STING activation is often associated with both NF-κB and IRF3 activation, several reports have suggested that interfering with proper translocation of STING from the ER to Golgi compartments can selectively stimulate the NF-κB pathway^48,49^. To test whether this is the case in SARS-CoV-2 infected cells, we determined the localization of STING relative to Golgi markers by microscopy. Consistent with previous reports, in cells transfected with dsDNA, we observed STING translocation to the Golgi compartment (Supplementary Fig. 4l)^50^. No significant colocalization of Golgi markers and STING was observed in either mock or SARS-CoV-2 infected cells, suggesting that STING translocation may be impaired (Fig. 3h, i). Moreover, we found that clusters of STING in SARS-CoV-2 infected cells co-localized with viral nucleocapsid (N) protein but not with the ER mark PDI (Fig. 3j, k). Together, these results suggest that the cGAS-STING axis is activated in SARS-CoV-2 infection but leads to a specific NF-κB inflammatory response in infected cells, possibly due to altered translocation from the ER to the Golgi compartment.

### Inhibition of the cGAS-STING axis limits SARS-CoV-2-mediated inflammatory gene activation

To corroborate our data and to test if pharmacological inhibition of cGAS-STING activation can limit SARS-CoV-2 induced pro-inflammatory cytokine production, we examined the effects of specific STING inhibitors in infected cells. Two different STING inhibitors were used: the previously described H-151 compound^51^ and the compound VS-X4 that is in preclinical development and advancing towards IND-enabled studies for in-human trials. To initially validate functionality and specificity of VS-X4, cells were incubated with different concentrations of VS-X4 followed by stimulation with cGAMP (STING activation), dsDNA containing vaccinia virus-derived DNA motifs (VACV; cGAS/STING activation), 5′pppdsRNA (RIG-I activation), or poly(I:C) (TLR3 activation). Activation of an IRF3-driven response was determined by using a luciferase reporter assay (Supplementary Fig. 5a). Under these conditions, the IC50 values (50% reduction of IRF3 activation) were 0.01 µM (cGAMP), 0.05 µM (VACV), 1 µM (5′pppdsRNA), and 1 µM (poly (I:C)), respectively. To determine the specificity of both VS-X4 and H-151 STING inhibitors in lung epithelial cells, cells were incubated with VS-X4, H-151, or the TBK1 inhibitor amlexanox (AMX), all at ten times the IC50, followed by transfection with either herring DNA or poly(I:C). Both STING inhibitors reduced *IFIT1* transcriptional activation in herring DNA transfected cells and had limited effects in poly(I:C) transfected cells (Supplementary Fig. 5b). Importantly, no significant decrease in *IFIT1* mRNA levels were observed in control cells treated with STING inhibitors, while *IFIT1* transcripts were decreased in all samples treated with AMX (Supplementary Fig. 5b).

**Fig. 4:**
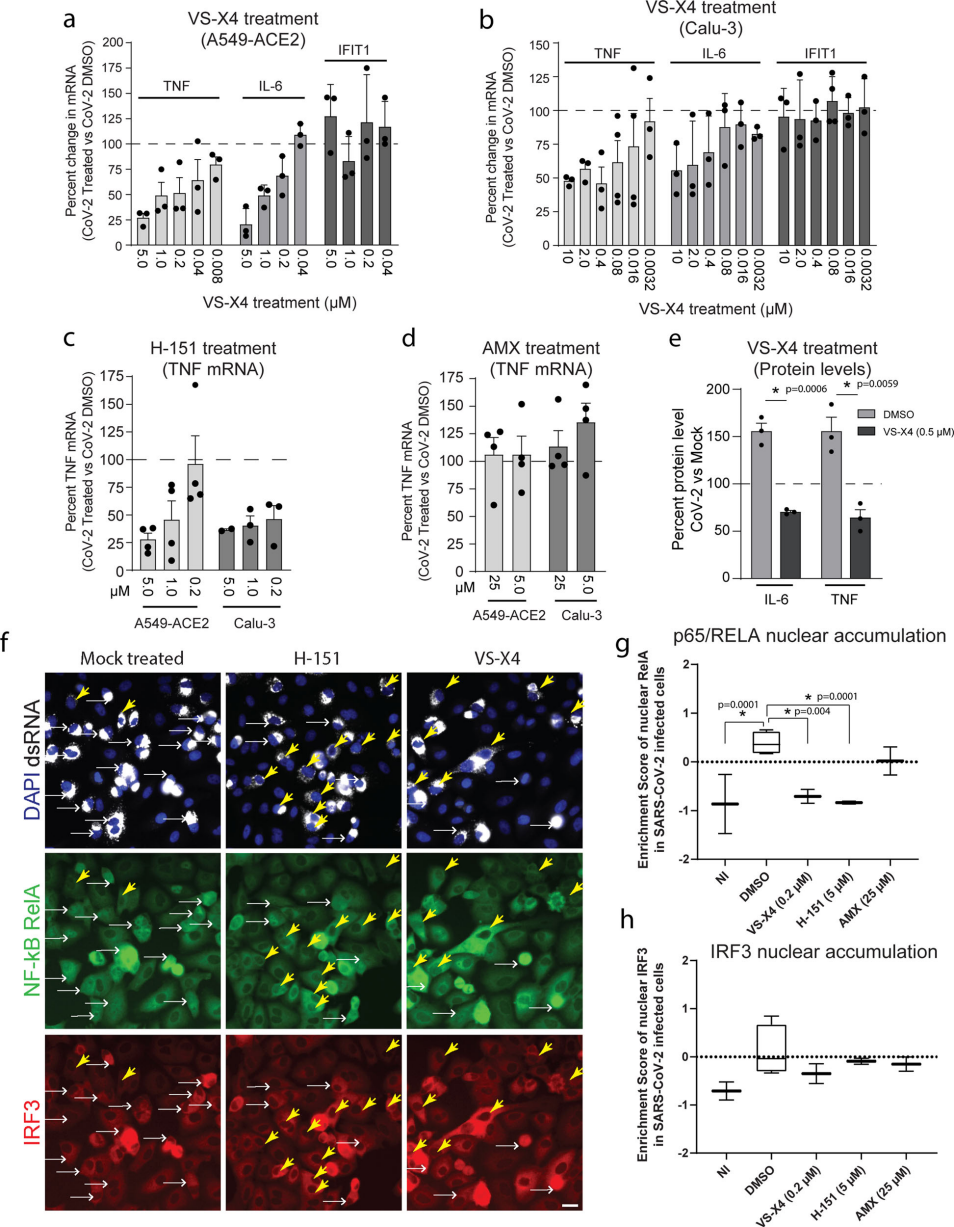
Pharmacological inhibition of cGAS-STING limits SARS-CoV-2 mediated inflammatory pathway activation. Cells were infected with SARS-CoV-2. One hour after infection cells were treated with the indicated drugs at the given concentrations. **a**–**d** Total RNA was isolated, and the indicated mRNA transcript levels were determined by RT-qPCR. Graphs show the average percent change and SEM for transcript levels compared to DMSO-treated cells for ≥3 independent experiments. **e** Protein levels from cell lysates were determined by western blotting. The graph shows the average percent change in protein levels in SARS-CoV-2 infected cells compared to mock. **f**–**h** A549-ACE2 cells were infected with SARS-CoV-2 for 1 h followed by treatment with the indicated drugs. 16 h after infection, cells were fixed and stained with antibodies directed against p65/RELA and IRF3. **f** Cells were imaged by confocal microscopy. White and yellow arrows indicate cells with positive or negative nuclear p65/RELA signals, respectively. Scale bars, 20 μm. **g**, **h** Graphs show the enrichment score for p65/RELA (**g**) or IRF3 (**h**) nuclear accumulation in cells treated with the indicated drugs. Graphs show the distribution of nuclear positive cells over three independent biological replicates. *N* > 4000 cells per condition. Box plots, box shows 25th–75th percentile; whiskers show min to max; line shows the mean value. Statistical significance was determined using one-way ANOVA with a Dunnett’s multiple comparison analysis. *represents statistical significance and exact *p* values are provided. For all panels, *n* ≥ 3 biological replicates.

Having confirmed the specificity of the STING inhibitors VS-X4 and H-151 in human lung cells, next we determined their effects in SARS-CoV-2 infection. One hour after infection, cells were treated with different concentrations of VS-X4, H-151, AMX, or DMSO, the latter two serving as controls. At 24 h post infection, we observed a significant decrease in the levels of *TNF* mRNAs in infected cells treated with VS-X4 or H-151, compared to DMSO or AMX treated cells, in both A549-ACE2 and Calu-3 cells (Fig. 4a–d). Additionally, VS-X4 treatment also significantly decreased virus-induced upregulation of *IL-6* and *IP-10*, while having limited effects on the IRF3 regulated genes *IFIT1* and *MX1* (Fig. 4a, b and Supplementary Fig. 5c–e). Consistent with the transcriptional analysis, the level of TNF and IL-6 protein upregulation induced by infection was also significantly decreased in VS-X4 treated cells compared to controls cells (Fig. 4e). Treatment of SARS-CoV-2 infected cells with STING inhibitors also caused a specific decrease in p65/RELA nuclear accumulation in SARS-CoV-2 infected cells while having no effect on IRF3 (Fig. 4f–h). Decreases in inflammatory gene activation or p65/RELA nuclear accumulation were not observed for AMX treated cells, corroborating that the TBK1-IRF3 pathway is not involved in this response (Fig. 4d, g). SARS-CoV-2 replication and spread as well as cell viability were not significantly affected at the effective concentration (Supplementary Fig. 5f–j). Importantly, drug treatments had no effect on the basal levels of *TNF*, *IL-6*, or *IFIT1* (Supplementary Fig. 5k, l). Together these results indicate that SARS-CoV-2-infection triggers the cGAS-STING pathway, leading to NF-κB-mediated induction of pro-inflammatory cytokines, and that this response can be controlled with STING inhibitors.

## Discussion

**Fig. 5:**
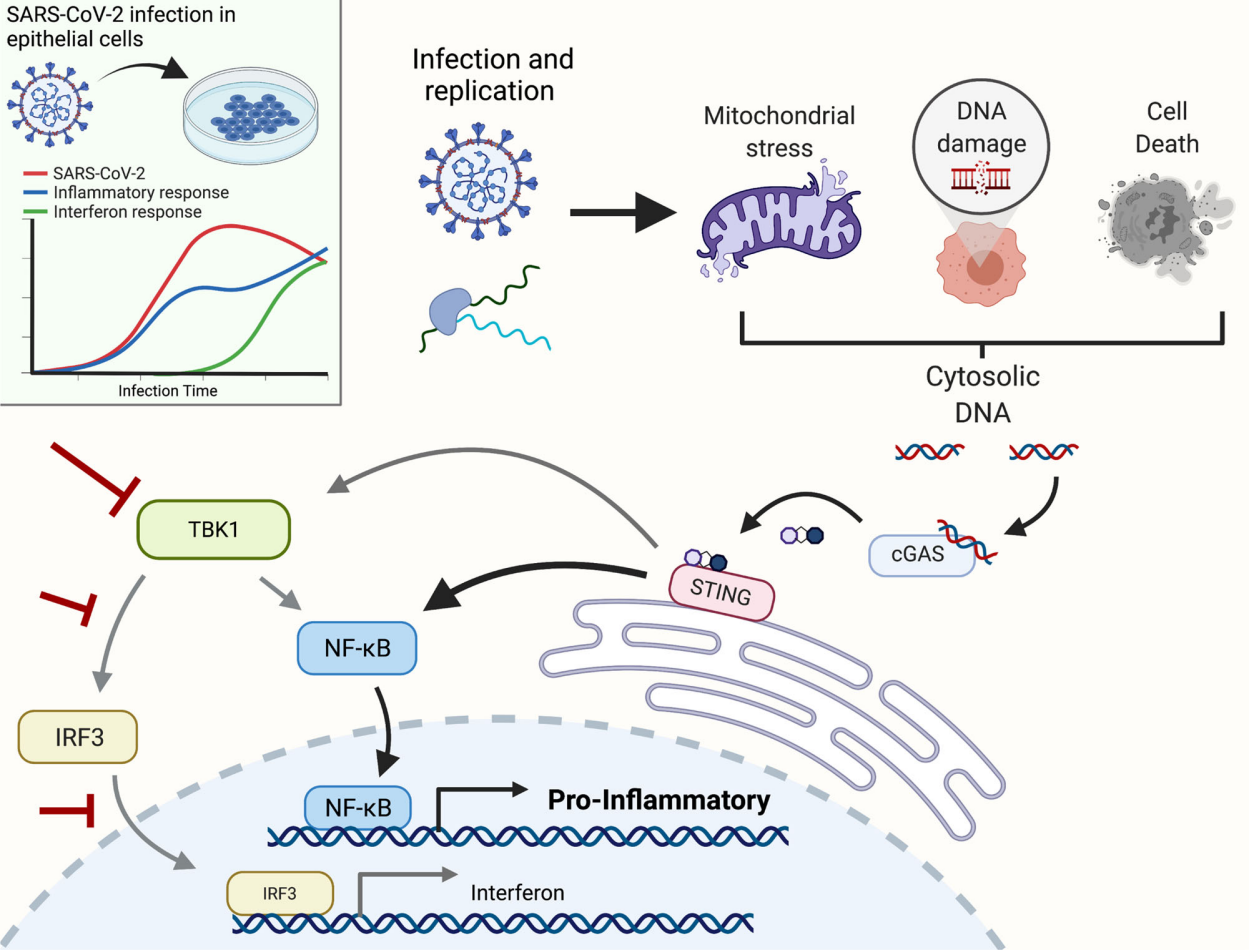
Summary figure. SARS-CoV-2 infection in epithelial cells leads to an early inflammatory response that correlates with virus RNA replication followed by a late interferon response that can be amplified by activated non-infected bystander cells. Virus infection and replication causes cellular stress that can lead to mitochondrial stress/damage, DNA damage, and cell death. Each of these responses can result in the activation of the cGAS-STING axis via cellular DNA leading either to direct activation of NF-κB or to TBK1-mediated activation of IRF3 and NF-κB. In SARS-CoV-2 infected cells, the TBK1 and IRF3 pathways are blocked by the action of several viral proteins. Therefore, STING activation leads to a predominant NF-κB response causing production and release or pro-inflammatory cytokines. Created with BioRender.com.

In this study, we combined transcriptional profiling and cytokine secretion analyses to characterize the pro-inflammatory response induced by SARS-CoV-2 infection and evaluated the virus-induced signaling pathways mediating this response. We report that both virus-induced transcriptional changes and cytokine profiles from two different infected epithelial cell lines are biased towards an inflammatory response, which is similar to the cytokine profiles observed in primary cells and with patient samples^2–5^. Most notable are the elevated levels of *IL-6* and *IP-10* as well as the transcriptional activation of *TNF* and TNF activated genes. Similar increases in *IL-6* and *IP-10* have been reported in ex vivo lung samples infected with SARS-CoV-2 and IL-6 and TNF have been reported as important factors mediating severe COVID-19^52–54^. These findings indicate a role for infected epithelial cells in contributing to initial hyper-inflammatory responses described for patients suffering severe COVID-19. This pro-inflammatory response in infected epithelial cells is initiated by activation of NF-κB with a concurrent robust block of the IRF3 and IFN pathways. We further demonstrate that this activation of NF-κB is not mediated by the expected viral RNA recognizing receptors of the RLR or TLR family, but instead, SARS-CoV-2 infection leads to the activation of the cGAS-STING signaling axis. Putatively, this response leads to selective activation of NF-κB while parallel pathways block activation of the IRF3/IFN system (for a summary see Fig. 5). The upregulation of NF-κB-regulated pro-inflammatory cytokines, such as TNF and IL-6, can be efficiently blocked by the administration of pharmacological STING inhibitors.

Pro-inflammatory cytokine production is an important aspect of the innate immune response that is required to recruit professional immune cells to the site of infection and aid in the initiation of the adaptive immune response. This response, together with the activation of antiviral pathways, including type I/III IFNs, creates a potent antiviral environment. Our study, in combination with several parallel studies, indicates that SARS-CoV-2 infection induces a selective inflammatory response that can cause pathogenic inflammation without effectively controlling the virus. This imbalanced immune response initiated in infected lung epithelial cells results in an NF-κB-polarized response rather than a classic antiviral immune response (NF-κB, IRF3/7, and IFN signaling), which is likely amplified by immune cells to produce the cytokine storm symptoms associated with COVID-19^3,12,55–57^. The levels of cytokine release we observe from infected lung epithelial cells are sufficient to activate downstream immune cells such as macrophages and neutrophils^30–32^. Indeed, a similar imbalanced immune response has been observed in SARS-CoV-2 infected hamsters, where high viral loads in the lower respiratory tract cause increased pro-inflammatory cytokine productions with limited IFN/ISG responses^34^. Moreover, this pro-inflammatory response led to the recruitment of macrophages and neutrophils, which correlated with cell death and lung pathology. Taken together with data from in vivo models, our observations indicate that infected cells, which are primarily lung epithelial cells, can initiate a pro-inflammatory response that likely contributes to immune cell recruitment. It is well conceivable that these signals are amplified by recruited myeloid cells leading to increased pathogenesis.

Our transcriptional analysis and cytokine profiles show that SARS-CoV-2 infection induces only a very low IFN response in infected lung epithelial cells at late time-points after infection. In addition to blocking IFN signaling, SARS-CoV-2 infection limits the activation of dsRNA sensing PRR pathways even when exogenously stimulated by poly(I:C) transfection. Corroborating our results, several parallel studies indicate that SARS-CoV-2 proteins interfere with multiple key steps in the RIG-I/MDA5 cellular immune responses. These include limiting MAVS activation by ORF9b or M protein, blocking TBK1 activation by nsp6, nsp13, and nsp15, perturbing IRF3 nuclear translocation by ORF6 as well as mechanisms to inhibit downstream IFN signaling by attenuating JAK/STAT signaling^20–27^. Despite this robust block in immune signaling, several groups have reported high levels of IFN production and IFN pathway activation through MDA5 specific recognition of viral RNA in lung epithelial cells or intestinal cells^14–17^. These differences in the activation of cell-intrinsic immune pathways might be explained by different experimental setups, notably the use of a low multiplicity of infection (MOI) and longer experiment duration which involves virus spread. In our study, which is comparable to several other reports, we used higher MOIs to synchronize the infection of lung epithelial cells, resulting in cell death starting at 15–16 h post infection^12,13,58^. In other systems with lower MOIs, the activation of IFN response was delayed, starting at 24 h post infection and peaking at 48–72 h post infection. In this case, the IFN pathway activation is not likely a direct response to the virus infection but rather an indirect response to virus spread and mediated by a bystander, rather than infected cells.

Further evaluation of the SARS-CoV-2 induced pro-inflammatory response showed a specific induction of NF-κB, but not of IRF3 or the subsequent IFN signaling. The selective activation of NF-κB, rather than a general block in all immune activation pathways, indicates a proviral role for NF-κB signaling. In addition to functions in inflammation, NF-κB is also important for cell survival and proliferation^59^. These NF-κB cell survival signals could be beneficial for the virus by promoting the vitality of cells in order to allow efficient and sustained virus replication and spread. Mechanisms for NF-κB pathway interference have been reported for numerous DNA and RNA viruses^60–62^. Selective modulation of the cGAS-STING pathways may allow SARS-CoV-2 to promote an NF-κB mediated cell survival signal while limiting ISG induction.

NF-κB can be activated through numerous immune or stress stimuli including the ER stress responses or increases in cytosolic reactive oxygen species, as well as through detection of cytosolic DNA released from the nucleus or mitochondria (reviewed in refs. ^8,63,64^). Our results indicate that inhibition of cGAS-STING activation leads to a 60–75% reduction in inflammatory gene activation following SARS-CoV-2 infection, suggesting that this pathway is a major contributor to NF-κB activation. Similar activation of cGAS-STING has been observed for other plus-strand RNA viruses including flaviviruses and both SARS-CoV and NL63 coronaviruses (reviewed in ref. ^9^). For other coronaviruses, STING activation is perturbed through the action of the viral PLpro leading to an inhibition of STING oligomerization and downstream activation of TBK1 and IRF3^65–67^. Our data suggest that the mechanisms for cGAS-STING modulation are different in SARS-CoV-2 infected cells, highlighting a major immunological difference between these related viruses. Of note, a parallel study to ours recently reported that interactions between the viral ORF3a protein and STING cause a block in STING-mediated NF-κB activation^68^. However, their study relied on a constitutively active cGAS/STING overexpression system outside the context of virus infection. Moreover, it has been shown that ORF3a primarily localizes to lysosomes where it functions to disrupt lysosomal acidification and facilitate viral egress^69^. It is therefore unclear whether this reported ORF3a-mediated counteraction of STING is a bona fide viral mechanism for limiting immune activation in infected cells.

Classical cGAS-STING induction activates not only NF-κB, but also TBK1 and IRF3 pathways. We envisage several mechanisms that could contribute to the selective NF-κB activation. First, the virus could actively block TBK1 activation in infected cells. Indeed, as stated above, protein interaction studies indicate that viral nsp6, nsp13, and nsp15 proteins interact with TBK1 or its adapter proteins and nsp6 has been shown to inhibit IRF3 activation^21,25^. Since the activation of NF-κB by STING can occur independently of TBK1 activation, the specific inhibition of TBK1 would not completely block NF-κB activation. Moreover, our results support a model where SARS-CoV-2 infection prevents activated STING from translocating from the ER to the Golgi^50^. Activation of STING at the ER has been shown to be sufficient for NF-κB activation, but not for TBK1 activation and the subsequent IRF3 phosphorylation^49,70^. It may be that fragmentation of the Golgi by SARS-CoV-2 infection leads to an impairment of STING translocation to the ERGIC^28^. Alternatively, SARS-CoV-2 proteins could actively block cGAS-STING translocation. We observed colocalization between STING and N protein in infected cells and others have reported interactions between STING and ORF3a, both suggesting a direct role for viral proteins manipulating the cGAS/STING pathway^68^. A similar mechanism has been suggested for murine cytomegalovirus, where viral m152 protein associates with STING and limits exit from the ER, thereby promoting an NF-κB specific response^48^.

The activation of NF-κB through cGAS-STING does not exclude other sources of NF-κB activation. Indeed, we observed increases in FOS/JUN and ATF3 mRNA levels in infected cells suggesting activation of multiple cell stress pathways^71^. Moreover, pharmacological inhibition of STING did not completely block pro-inflammatory cytokine gene upregulation, further indicating a role for other sources of activation. We speculate that therapeutic inhibition of multiple NF-κB activation pathways could serve to further reduce pro-inflammatory responses in SARS-CoV-2 infected cells. In addition to other immune modulators that are currently being used or clinically evaluated (e.g., IL-6 inhibitors or corticosteroids)^72–76^, our results indicate that disease severity might be suppressed at the epithelial cell level through blocking NF-κB mediated inflammatory responses^57^. In this respect, NF-κB inhibitors analogous to CAPE or parthenolide, which have been shown to prolong the survival of SARS-CoV infected mice^77^, might help to reduce the disease burden imposed by COVID-19.

## Methods

### Cell lines, culture conditions, and viruses

Calu-3 and A549 cells were cultured in Dulbecco’s Modified Eagle’s Medium (DMEM) supplemented with Glutamax (Gibco), 10% fetal bovine serum, 100 U penicillin/ml, 100 μg streptomycin/ml, 2 mM l-glutamine, and nonessential amino acids. A549 cells, a commonly mislabeled cell line, were used in this study to evaluate the role of SARS-CoV-2 in lung epithelial cells, which are the initial site of virus infection in humans. All cell lines used in this study tested negative for mycoplasma contamination. A549 cells stably expressing ACE2 and the SARS-CoV-2 reporter construct were created by lentiviral transduction. To produce lentivirus particles, HEK-293T cells were transfected with pCMV-Gag-Pol, pMD2-VSV-G (kind gifts from Didier Trono, EPFL, Lausanne, Switzerland), and a pWPI vector encoding the gene of interest. Transfections were done using polyethyleneimine and lentivirus particles were harvested and filtered through a 0.45 µm pore-size filter. A549 cells were inoculated with the viral supernatant overnight and the next day antibiotic selections were applied. Neomycin (500 µg/ml) and Puromycin (2 µg/ml) antibiotics were used for ACE2 and SARS-CoV-2 reporter expressions, respectively. Viruses used are SARS-CoV-2- BavPat1/2020 strain (kindly provided by Christian Drosten through the European Virus Archive) and ZIKV H/PF/2013 (GenBank accession number KJ776791.2),

### Expression constructs, transduction, and transfection

For stable cell line production, cDNAs encoding for STING or cGAS were amplified with primers containing the ATTB recombination overhangs, followed by recombination into the pDONR207 entry vector using the BP recombination reaction (Invitrogen). From the entry vectors, sequences were transferred to either the previously described pWPI-nHA or pWPI expression vectors, containing the ROSA26 promoter sequence for low expression levels in mammalian cells, using the Gateway LR clonase protocol^78^. Delivery of expression constructs to obtain stable cells lines was done through transduction with lentiviruses. For the production of lentivirus stocks, sub-confluent 293 T cells were transfected with packaging plasmids pCMV-Gag-Pol and pMD2-VSV-G (kind gifts from Didier Trono, EPFL, Lausanne) and the specific expression plasmids. A549-ACE2 cells were transduced with lentivirus particles and, one day after transduction, cells were incubated with 1 µg/mL puromycin to select cells containing the integrated proviral DNA.

For siRNA-mediated depletion experiments, siRNA pools for cGAS or nontarget control siRNAs were purchased from siTOOLS Biotech GmbH (Planegg). About 10 nM of each siRNA was transfected into A549-ACE2 cells using Lipofectamine RNAi-MAX transfection reagent following the manufacturer’s protocol. Three days after transfection, cells were infected with SARS-CoV-2 at an MOI of 2.0, and 16 h later total RNA was isolated from cells and analyzed by RT-qPCR.

### SARS-CoV-2 virus stock production

SARS-CoV-2 stocks were produced using the VeroE6 cell line. Passage 2 BavPat1/2020 (MOI: 0.01) strain was used to generate the seed virus (passage 3). After 48 h the supernatant was harvested, cell debris was removed by centrifugation at 1000 × *g* for 5 min, and the supernatant was filtered with a 0.45 mm pore-size filter. Passage 4 virus stocks were produced by using 500 μl of the seed virus (passage 3) to infect 9E + 06 VeroE6 cells. The resulting supernatant was harvested, filtered 48 h later as described above, and stored in aliquots at −80 °C. Stock virus titers were determined by plaque assay.

### RNA isolation and RT-qPCR

Total RNA was isolated from cells or supernatants using the NucleoSpin RNA extraction kit (Macherey-Nagel) according to the manufacturer’s specification. cDNA was synthesized from the total RNA using the high-capacity cDNA reverse transcription (RT) kit (ThermoScientific) according to the manufacturer’s specifications. Each cDNA sample was diluted 1:15 in nuclease-free H_2_O prior to qPCR analysis using specific primers and the iTaq Universal SYBR green mastermix (Bio-Rad). Primers for qPCR were designed using Primer3 software and include: SARS-CoV-2-ORF1 fwrd-5′-GAGAGCCTTGTCCCTGGTTT-3′, rev-5′-AGTCTCCAAAGCCACGTACG-3′; IFIT1 fwrd-5′-GAAGCAGGCAATCACAGAAA-3′, rev-5′-TGAAACCGACCATAGTGGAA-3′; IFIT3 fwrd-5′-GAACATGCTGACCAAGCAG-3′, rev-5′-CAGTTGTGTCCACCCTTCC-3′; TNF fwrd-5′-TAGCCCATGTTGTAGCAAACCC-3′, rev-5′-GGACCTGGGAGTAGATGAGGT-3′; GAPDH fwrd-5′-GAAGGTGAAGGTCGGAGTC-3′, rev-5′-GAAGATGGTGATGGGATTTC-3′; HPRT fwrd-5′-CCTGGCGTCGTGATTAGTG-3′, rev-5′-ACACCCTTTCCAAATCCTCAG-3′, IL-6 fwrd-5′-CCAGAGCTGTGCAGATGAGT-3′, rev-5′-ATTTGTGGTTGGGTCAGGGG-3′, TNFAIP3 fwrd-5′-CAGGACTTGGGACTTTGCGA-3′, rev-5′-GTGCTCTCCAACACCTCTCC-3′, cGAS fwrd-5′-GACCACCTGCTGCTCAGACT-3′, rev-5′-GTGCAGAAATCTTCACGTGCT-3′, ZIKV fwrd-5′-AGATGAACTGATTGGCCGGGC-3′, rev-5′-AGGTCTCTTCTGTGGAAATA-3′, MX1 fwrd-5′-ACCATTCCAAGGAGGTGCAG-3′, rev-5′-TGCGATGTCCACTTCGGAAA-3′. To obtain the relative abundance of specific RNAs from each sample, cycle threshold (ct) values were corrected for the PCR efficiency of the specific primer set and normalized to hypoxanthine phosphoribosyltransferase 1 *(HPRT*) transcript levels.

For microarray chip analysis total RNA was extracted from cells and hybridized on an Affymetrix Clariom S human array performed by the Microarray Unit of the Genomics and Proteomics Core Facility at the German Cancer Research Center (DKFZ). Labeling was done using the Thermo Fisher Scientific (Affymetrix) Gene Chip WT PLUS Reagent to generate labeled ss-cDNA from input amounts of 50 ng total RNA. Hybridization was done according to the manufacturer’s protocol for Thermo Fisher Scientific (Affymetrix) Gene Chip WT PLUS Reagent Kit. 5.5 µg of fragmented and labeled ss-cDNA were hybridized for 17 h at 45 °C on Thermo Fisher Scientific (Affymetrix) human Clariom S Arrays. Chip scanning Gene Expression Microarrays were scanned using the Affymetrix GeneChip Scanner 3000 according to GeneChip Expression Wash, Stain and Scan Manual for Cartridge Arrays

### Data analysis for microarray

Raw, analyzed, and metadata as well as the code used during analysis are available upon request.

First, data were collected for all samples after Robust Multi-Array Average (RMA) quantile normalization with R using the function “normalize.quantiles” from Bioconductor package “preprocessCore” for probe set equalization. Second, data were log-transformed and PCA including all samples was performed using R/prcomp (R version 4.0.0). The rotation for each sample is shown. After PCA quality control and check for equal distribution of log-transformed probe intensities, data was gathered, and time-points were pooled as “early” (4 and 8 h time-point) or “late” (12 and 24 h). R/limma’s lmFit, eBayes, and topTable functions were then used with a model “matrix of expression ~ treatment + time” (limma version 3.40.6,^79^), to estimate base mean expression and differential expression for the contrast infected vs mock treatment. This analysis was performed individually for each cell line as differences between the lines would have obscured a model by driving the variance, as apparent in the PCA analysis. R/limma’s topTable function employs Benjamini–Hochberg correction for multiple testing on all *p* values. Genes were called significant if their adjusted *p* value was smaller than 0.1 (false discovery rate, FDR < 10%).

Gene set enrichment analysis was performed according to Subramanian et al.^80^. We use the practical R implementation “fgsea”^81^ and the hallmark pathway gene set published by Liberzon et al.^82^. The barcode plot implementation was inspired by Zhan et al.^83^.

### Antibodies

Primary antibodies and specific dilutions used for western blot or immunofluorescence included: Mouse anti-dsRNA J2 (Scicons: 10010500, IF-1:1000); Mouse anti-SARS-CoV-2 N protein (Sino Biological: 40143-MM05, IF - 1:1000; WB-1:1000); Rabbit anti-SARS-CoV-2 Spike protein (Abcam: ab252690, WB- 1:1000); Rabbit anti-IRF3 (Cell Signaling Technology: 11904 S, IF - 1:400); Mouse anti-P65/RELA (Santa Cruz: sc-8008, IF - 1:100); Rabbit anti-cGAS (Atlas Antibodies: HPA031700, IF-1:100); Rabbit anti-STING (Atlas Antibodies: HPA038534, IF-1:100); Mouse anti-dsDNA (Abcam: ab27156, IF-1:2000); Rabbit anti-p65/RELA (Cell Signaling: L8F6, WB-1:1000); Rabbit anti-phospho-p65/RELA (Cell Signaling: 3033, WB-1:1000); Rabbit anti-IkB (Cell Signaling: 9242 s, WB-1:1000); Sheep and-TGN46 (Biorad:AHP500G, IF-1:200); Mouse anti-Actin (Sigma Aldrich: A5441, WB-1:5000); Rabbit anti-HA (Thermo Fisher PA1-985 IF-1:500); Rabbit anti-pIRF3 (Cell Signaling: 4947, WB-1:1000); Mouse anti-PDI (Thermo Fisher: MA3-019, IF-1:200); Mouse anti-IL-6 (R&D systems: MAB2061-100, Neutralization 1:1000); Goat anti-TNF (R&D systems: AF-410-NA, Neutralization 1:5000); rabbit anti-IL-6 (Thermo: P620, WB-1:500); Rabbit anti-TNF (Thermo: AMC3012, WB-1:500); Mouse anti-LaminA/C (Santa Cruz: sc-7292, WB-1:1000).

Secondary antibodies used for western blot included Goat anti-rabbit IgG-HRP (Sigma Aldrich A6154, 1:2000), Goat anti-mouse IgG-HRP (Sigma Aldrich A4416, 1:5000). Secondary antibodies for immunofluorescence included: Alexa Fluor 488 donkey anti-rabbit IgG (Thermo Fisher A-21206), Alexa Fluor 488 donkey anti-mouse IgG (Thermo Fisher A-21202), Alexa Fluor 488 donkey anti-mouse IgG2a (Thermo Fisher A-21131), Alexa Fluor 568 donkey anti-rabbit IgG (Thermo Fisher A-10042), Alexa Fluor 568 donkey anti-mouse IgG (Thermo Fisher A-10037), Alexa Fluor 568 donkey anti-mouse IgG1 (Thermo Fisher A-21124), Alexa Fluor 647 donkey anti-rabbit IgG (Thermo Fisher A -31573), Alexa Fluor 647 donkey anti-mouse IgG (Thermo Fisher A-31571). ALL Alexa fluor secondary antibodies were used at 1:1000.

### Immunofluorescence analysis

After infection with SARS-CoV-2 cells were fixed with 6% formaldehyde solution, washed twice with phosphate-buffered saline (PBS), and permeabilized with 0.2% Triton X-100 in PBS. Next, the Triton X-100 solution was replaced with 2.5% (w/v) milk solution (in PBS), and cells were blocked for 1 h at room temperature. Primary antibodies were diluted in 2.5% milk solution and samples were incubated with primary antibodies for 1 h. After washing three times with PBS, samples were incubated with Fluorophore-conjugated secondary antibodies, diluted in milk solution, for 30 min. After washing three times with PBS samples were mounted in Fluoromount G solution containing DAPI (Southern biotech) for DNA staining. Microscopic analyses were conducted with a Nikon Eclipse Ti microscope, a Nikon Andor spinning disk confocal microscope (Nikon, Tokyo, Japan), or a Leica SP8 confocal microscope (Leica) for the subcellular localization analyses.

For quantification of the nuclear translocation of NF-κB p65/RELA or IRF3, nuclei were first segmented using the DAPI signal. Second, the segmented nucleus was dilated and the dilated nucleus was subtracted by the original nucleus mask to detect perinuclear fluorescent signal. To determine SARS-CoV-2 infected cells, dsRNA intensity was measured within the perinuclear area. Using the CellProfiler Analyst image analysis software, a semi-supervised machine learning-based classifier was trained to identify a class that defines the cells that have nuclear translocated either NF-κB p65/RELA or IRF3^84^. To determine the enrichment score of nuclear-translocated class in a certain sample, the probability of the presence of this class in relation to the total cells in the sample was calculated and the data normalized to the control sample was plotted^85^. The scripts, training sets, and images are available on request.

### Cytokine neutralization assay

To determine the neutralization capacity of IL-6 and TNF antibodies, A549-ACE2 cells seeded into 24-well plates were incubated with anti-IL-6 (500 ng/mL) or anti-TNF (40 ng/ml) neutralizing antibodies for 30 min. Recombinant IL-6 (1 ng/mL) or TNF (0.1 ng/mL) was added to each well containing A549-ACE2 cells and neutralizing antibodies. Six hours after cytokine addition, cells were harvested and mRNA transcript level changes were evaluated by RT-qPCR. For virus infection experiments, A549-ACE2 or Calu-3 cells seeded into 24-well plates were incubated with IL-6 (500 ng/mL) or TNF (40 ng/ml) neutralizing antibodies for 30 min. SARS-CoV-2 was then added to each well at an MOI of 5. Sixteen hours after infection, cells were harvested and mRNA transcript level changes were evaluated by RT-qPCR.

### Poly(I:C) and herring DNA transfection

For Calu-3 or A549-ACE2 cell stimulation, cells were transfected with the indicated amount of poly(I:C) using lipofectamine 2000 reagent as per the manufacturer’s protocol. Sixteen hours after transfection, total RNA was isolated, and RT-qPCR was used to determine transcript levels as described above.

For transfection in SARS-CoV-2 infected cells (Fig. 5 and Fig. S3), cells seeded in 24-well plates were infected with SARS-CoV-2 at MOI = 5 for 6 h. Cells were then transfected with poly(I:C) or herring DNA (500 ng/well) using lipofectamine 2000 reagent as per the manufacturer’s protocol. Six hours after transfection, cells were either fixed with 4% paraformaldehyde and processed for immunofluorescence, or total RNA was isolated for RT-qPCR analysis as described above.

### Western blot analysis and subcellular fractionation

Infected and mock cells were washed with PBS and lysed with 100 µl of sample buffer (120 mM Tris-HCl [pH 6.8], 60 mM SDS, 100 mM DTT, 1.75% glycerol, 0.1% bromophenol blue) supplied with 1 µl of benzonase (Millipore: 70746-3) to remove contaminating nucleic acids. Denaturation of the samples was achieved by incubation at 95 °C for 3 min. After SDS-PAGE, proteins were blotted onto PVDF (polyvinylidene fluoride) membranes and blocking was done with 3% (w/v) BSA in Tris-buffered saline (TBS) for 1 h at room temperature. Membranes were incubated with primary antibodies, diluted in 3% BSA in TBS, for 1 h, and washed three times for 10 min each with TBS-T (TBS supplied with 0.1% Tween 20). Horseradish peroxidase (HRP)-conjugated secondary antibodies were diluted in 5% (w/v) milk in TBS-T and membranes were incubated for 1 h at room temperature. After washing three times with TBS-T for 10 min, membranes were developed with the Western Lightning Plus-ECL reagent (Perkin Elmer: NEL105001EA). A ChemoCam Imager 3.2 (Intas Science Imaging Instruments GmbH, Göttingen, Germany) was used to visualize the signals that were quantified using the ImageJ (FiJi) software package.

Nuclear and cytosolic fractionation experiments were performed as previously described^86^. Specifically, Calu-3 or A549-ACE2 cells were seeded into 10-cm diameter dishes and infected with SARS-CoV-2 for 16 h. Cells were washed once in ice-cold PBS and scraped in 1 ml of ice-cold PBS. Cells were centrifuged at 21,000 × *g* for 10 s, the supernatant was removed, and cells were lysed in 900 µl lysis buffer (0.1% NP40 in PBS). Three hundred µl was removed for whole-cell lysate samples. The remaining 600 µl was centrifuged at 21,000 × *g* for 10 s and 300 µl of the supernatant was taken for the cytosolic fraction. The remaining supernatant was removed and the pellet was resuspended in 1 ml lysis buffer followed by centrifugation at 21,000 × *g* for 10 s. The pellet was harvested as the nuclear fraction. Laemmli buffer was added to all samples that were separated by SDS-PAGE and analyzed by western blot.

### cGAMP ELISA

For cGAMP ELISA analysis, infected and mock cells were washed with PBS followed by lysis using M-PER Mammalian Protein Extraction Reagent (Thermo Fisher). Lysates were stored at −80 °C until use. Total protein in each sample was determined using a Bradford assay. The ELISA analysis was performed using the Caymen Chemical cGAMP ELISA kit as per the manufacturer’s protocol. Cellular cGAMP levels were corrected for total protein levels in each sample.

### Plaque assay and CPE assay

2.5E + 06 VeroE6 cells were seeded into each well of a 24-well plate. On the next day, cells were infected with serial dilutions of SARS-CoV-2 for 1 h. Afterward, inoculum was removed and replaced with serum-free DMEM containing 0.8% carboxymethylcellulose. At 72 h post infection, cells were fixed with 5% formaldehyde for 1 h followed by staining with 1% crystal violet solution. Plaque forming units per ml (PFU/ml) were calculated by manual counting of the viral plaques.

### Drug treatments

Compounds H-151 (InvivoGen) and amlexanox (Abcam) were dissolved in DMSO to create stock solutions. VS-X4, a small molecule heterocycle, was designed and synthesized at Spring Bank Pharmaceuticals, Inc. To assess the STING-mediated inhibition of IRF3 and NF-κB by VS-X4, THP1-Dual-WT cells (InvivoGen) that allow the simultaneous study of the NF-κB pathway and the IRF3 pathway by two different reporters, were seeded into 96-well plates. Cells were pretreated with various concentrations of VS-X4 for 2 h, followed by 18 h-stimulation with 2′,3′-cGAMP (0.5 µM) that was mixed with lipofectamine 2000. The levels of IRF activity were determined using the Quanti-Luc assay for IRF3 and IC_50_ values were calculated against those obtained with DMSO-treated control cells using the Excel add-in Xlfit software package. In multiple experiments, the IC_50_ for IRF3 inhibition by VS-X4 ranged from 0.01 to 0.04 µM. Inhibition of NF-κB activity by VS-X4 was evaluated using dsDNA. For this, THP1-Dual-WT cells were seeded into 96-well plates and pretreated with various concentrations of VS-X4 for 2 h, followed by 18 h-stimulation with herring DNA (1 µg/ml) that was mixed with lipofectamine 2000. The levels of NF-κB in VS-X4-treated and DMSO-treated cell culture supernatants were determined using the Quanti-blue assay (Invivogen), and IC_50_ values were determined by using Xlfit.

### Cytokine profiling of patient samples

Patient sera were collected and stored at −80 °C until cytokine measurement. All material was obtained after approval by the Ethics Committee of the Medical Faculty Heidelberg (number S-148/2020, medical ethics committee of the University of Heidelberg); written consent was obtained from all patients prior to analysis.

Blood serum samples were evaluated for cytokine levels and compared to the cytokines secreted from infected culture cells. Serum was separated from clotted blood fraction by centrifugation at 1500 × *g* for 10 min at 4 °C. Analyses were performed with the Extended Bio-Plex Pro Human Cytokine 48-Plex Screening Panel (Bio-Rad, Munich, Germany)^87^ and using a two-laser reader allowing simultaneous quantification of cytokines and chemokines. Standard curves and concentrations were calculated with the Bio-Plex Manager software using the five-parameter logistic plot regression formula. The detection sensitivity of all analyses ranged from 2 pg/mL to 30 ng/mL. Alternatively, samples were analyzed by flow cytometry (BD LSRFortessa from BD Biosciences) using the LEGENDplex antiviral response panel (BioLegend) or the Meso Scale Discovery (MSD) V-plex assay according to the manufacturer’s instruction.

### Statistics and reproducibility

Statistical analysis was performed as stated in the methods of figure legends on at least three independent biological replicates. Unless otherwise stated, all experiments were performed with *n* ≥ 3 biological replicates.

### Reporting Summary

Further information on research design is available in the Nature Research Reporting Summary linked to this article.

## Supplementary information

Supplementary Information

Description of Additional Supplementary Files

Supplementary Data 1

Reporting Summary

## Data Availability

Microarray data used to support this study is deposited in Gene Expression Omnibus database repository with the accession number GSE189086, (https://www.ncbi.nlm.nih.gov/geo/query/acc.cgi?acc=GSE189086)^88^. The scripts to reproduce the processed data and all figures is available online (https://github.com/boutroslab/Supp_Neufeldt_2021). Data used to produce all graphs in the manuscript can be found in Supplementary data 1. The raw western blots used to make panels in Supplementary Fig. 3 can be found in Supplementary Fig. 6. The remainder of the data that support the findings of this study are available from the corresponding authors upon reasonable request.
