# Supplementary material for: SARS-CoV-2 infection induces a pro-inflammatory cytokine response through cGAS-STING and NF-κB
Source: Commun Biol. 2022 Jan 12;5:45. doi: 10.1038/s42003-021-02983-5 (PMC8755718; doi:10.1038/s42003-021-02983-5)
Supplement: Supplementary file 1 — Supplementary Information [file 42003_2021_2983_MOESM1_ESM.pdf]

Supplementary Figures

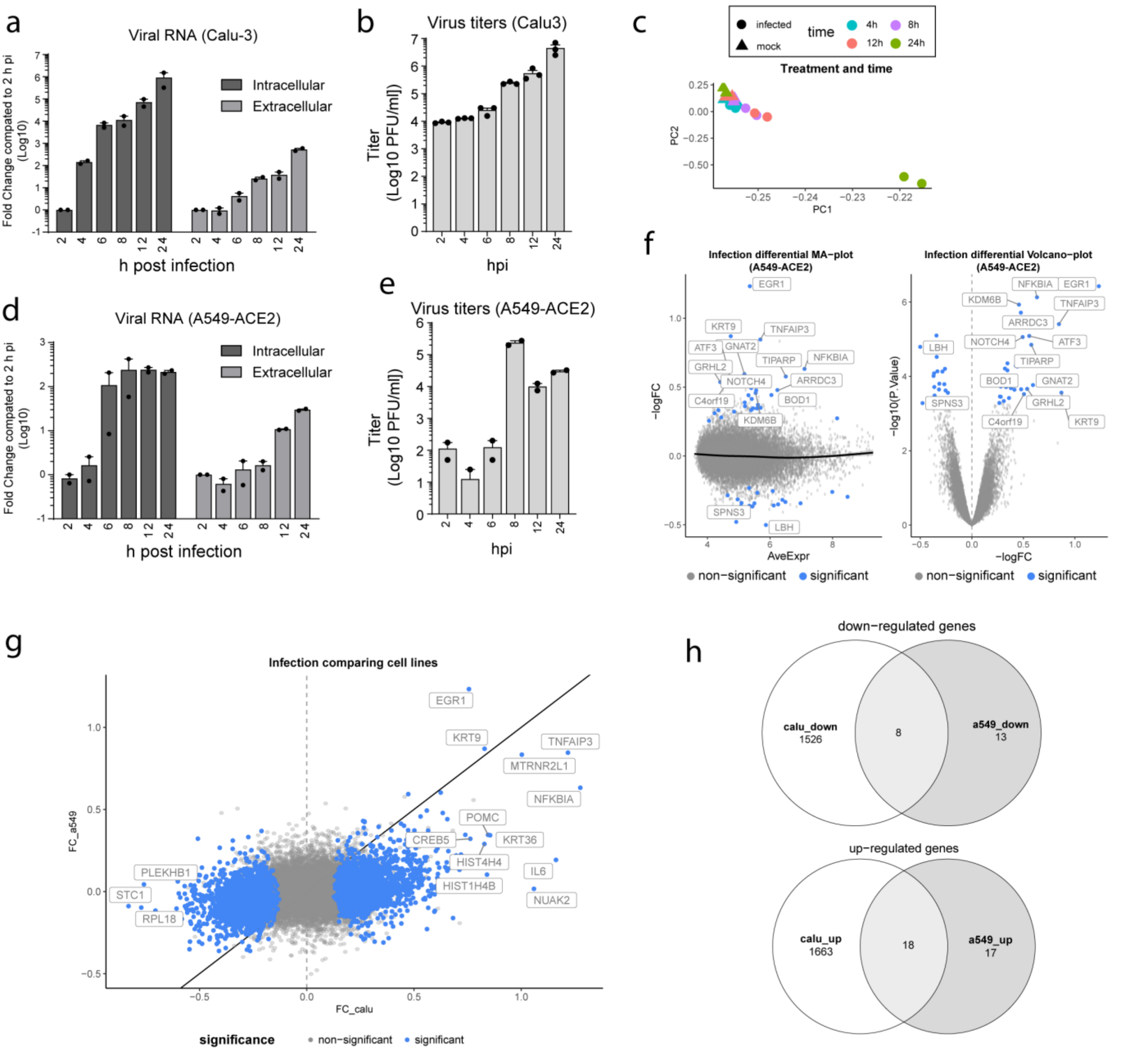

Supplementary Figure 1 | a-b, Calu-3 cells were infected with SARS-CoV-2 (MOI=1). At the indicated times after infection samples were harvested for analysis of intra- and extra-cellular viral RNA (a), titer of infectious virus released into culture supernatant (b). **a,** Total RNA was isolated from supernatants or cells at the given time-points and virus RNA levels were determined using RT-qPCR with ORF1a specific primers detecting only the viral RNA genome, but not subgenomic mRNAs. Graphs show the average fold change and SEM for each time-point compared to the 2 h time-point. Intracellular viral RNA levels were corrected for total cell numbers using HPRT as a standard. **b**, Infectious virus titers were determined using a PFU assay; graph shows the mean and SEM PFU/mL for each time-point. **c,** Principal component analysis on microarray data set. Calu-3 cells show separation between infection and mock but the A549-ACE2 cells have less separation due to lower levels of infection. **d-e,** A549-ACE2 cells were infected with SARS-CoV-2 (MOI=1). At the indicated times after infection samples were harvested for analysis of intra- and extra-cellular viral RNA (d), titer of infectious virus released into culture supernatant (e). **d,** Total cellular RNA was isolated from supernatants or cells at the given time-points and virus RNA levels were determined

using RT-qPCR with ORF1a specific primers detecting only the viral RNA genome, but not subgenomic mRNAs. Graphs show the average fold change and SEM for each time-point compared to the 2 h time-point. Intracellular viral RNA levels were corrected for total cell numbers using HPRT as a standard. **e**, Infectious virus titers were determined using a PFU assay; graph shows the mean and SEM PFU/mL for each time-point. **f,** MA-plots and Volcano-plots of transcriptional changes in A549-ACE2 cells highlighting differentially expressed genes. Blue dots represent significant changes as determined by R/limma with a Benjamini Hochberg adjusted p-value smaller than 0.1. Left plot (MA-plot) shows log2 fold change on the y-axis vs mean normalized expression on the x-axis. Right plot (volcano-plot) shows the significant hits considering both infection and changes over time (x-axis = log2 fold change; y-axis = -log10 p-value; top 15 significant genes marked). **g,** Plot comparing enriched genes between the two cell lines. **h,** Venn diagram of significantly (FDR < 10 %) upregulated (top) or downregulated (bottom) genes comparing Calu-3 and A549-ACE2 cells. For transcriptome analysis, n=2 biological replicates

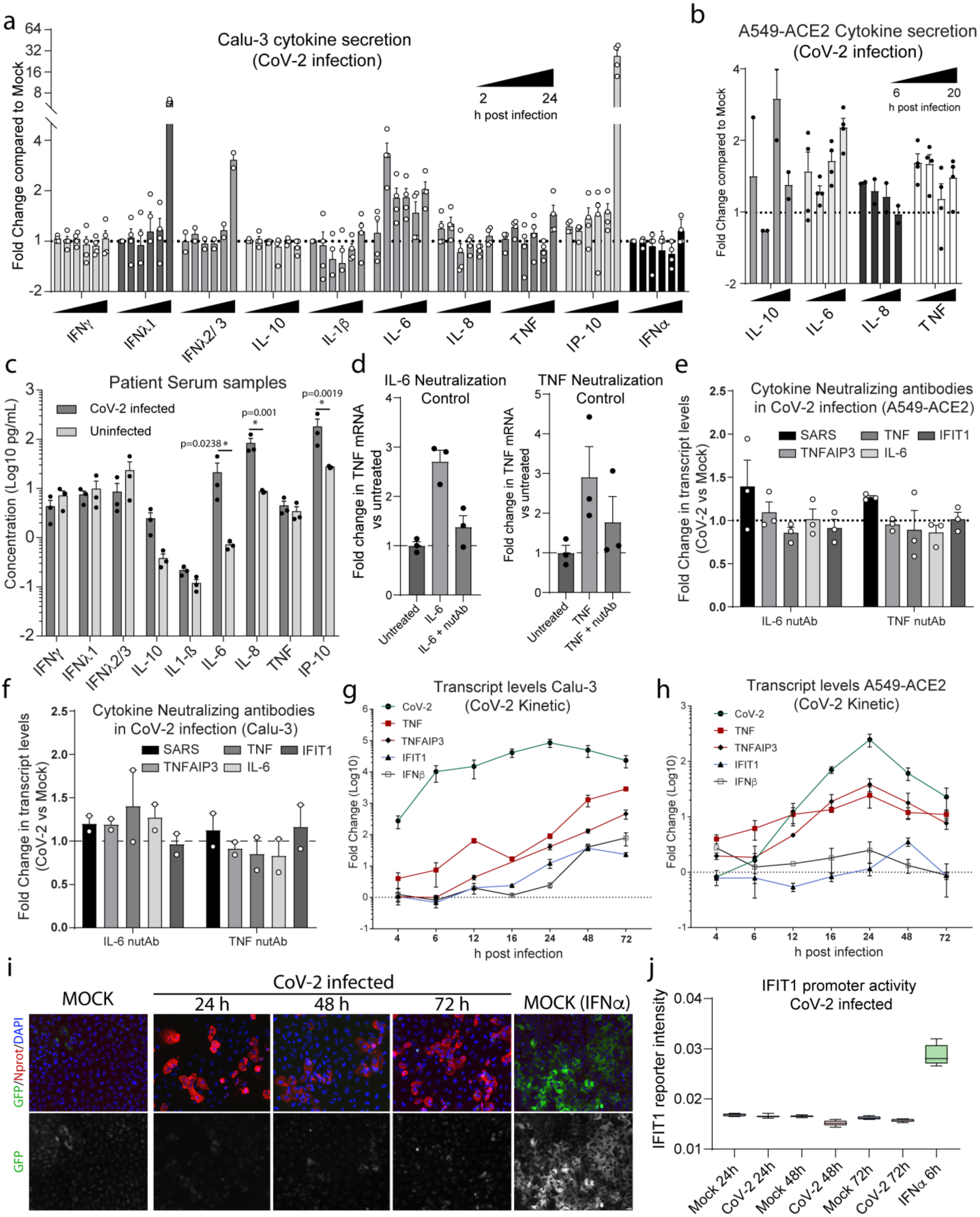

Supplementary Figure 2 | a, Calu-3 cells were infected with SARS-CoV-2. At 2 h, 4 h, 6 h, 8 h, 12 h, and 24 h post infection, Calu-3 cell culture supernatants were harvested from mock cells and infected cells,

treated with beta-propiolactone and cytokine profiles were determined by flow cytometry using the LGENDplex antiviral response panel. Values obtained for each infected sample were corrected for the corresponding value of the mock sample. Graph shows the mean and SEM of fold change for each cytokine compared to mock sample at each time-point. The y-axis scale is displayed in log2 increments. **b,** A549-ACE2 cells were infected with SARS-CoV-2. At 6 h, 10 h, 16 h, and 20 h post infection, cell culture supernatants were harvested from mock cells and infected cells, treated with beta-propiolactone and cytokine profiles were determined using the mesoscale MSD V-PLEX cytokine panel. Values obtained for each infected sample were corrected for the corresponding value of the mock sample. Graph shows the mean and SEM of the fold change for each cytokine compared to mock sample at each time-point. The y-axis scale is displayed in log2 increments. **c,** Serum samples were taken from patients with severe COVID-19 symptoms. Samples were treated with beta-propiolactone to inactivate virus while retaining antigenicity. The cytokine profiles were determined using the mesoscale MSD V-PLEX cytokine panel. Graphs show the mean concentration for each cytokine (pg/mL) detected in the various samples. Statistical significance was determined using students t-test. * represents statistical significance and exact p values are provided. **d,** A549-ACE2 cells were incubated with IL-6 (left) or TNF (right) neutralizing antibodies for 30 min. Recombinant IL-6 or TNF were then added to cells for 6 h and the levels of TNF mRNA transcripts were determined by RT-qPCR. Graphs show the mean fold change in mRNA levels compared to untreated cells. **e-f,** A549-ACE2 or Calu-3 cells were incubated with IL-6 or TNF neutralizing antibodies for 30 min. Cells were then infected with SARS-CoV-2 in the presence of neutralizing antibodies for 16 h and the levels of indicated mRNA transcripts were determined by RT-qPCR. Graphs show the mean fold change in mRNA levels compared to mock infected cells. **f,** n=2 biological replicates. **g-h,** Calu-3 or A549-ACE2 cells were infected with SARS-CoV-2 for the indicated times. The levels of indicated mRNA transcripts were determined by RT-qPCR. Graphs show the mean fold change in mRNA levels compared to mock infected cells. **i-j,** A549-ACE2 cells with an IFIT1 promoter GFP reporter (A549-IFIT1deGFP) were infected with SARS-CoV-2 for the indicated time or incubated with IFNα for 6 h. Cells were then fixed and stained for SARS-CoV-2 N protein. **i,** Panel shows representative images from each time point. Scale bar represents 50 μm. **j,** Graph shows the mean GFP fluorescence intensity for Mock and Infected cells at the indicated time points. Unless otherwise stated, n≥3 biological replicates.

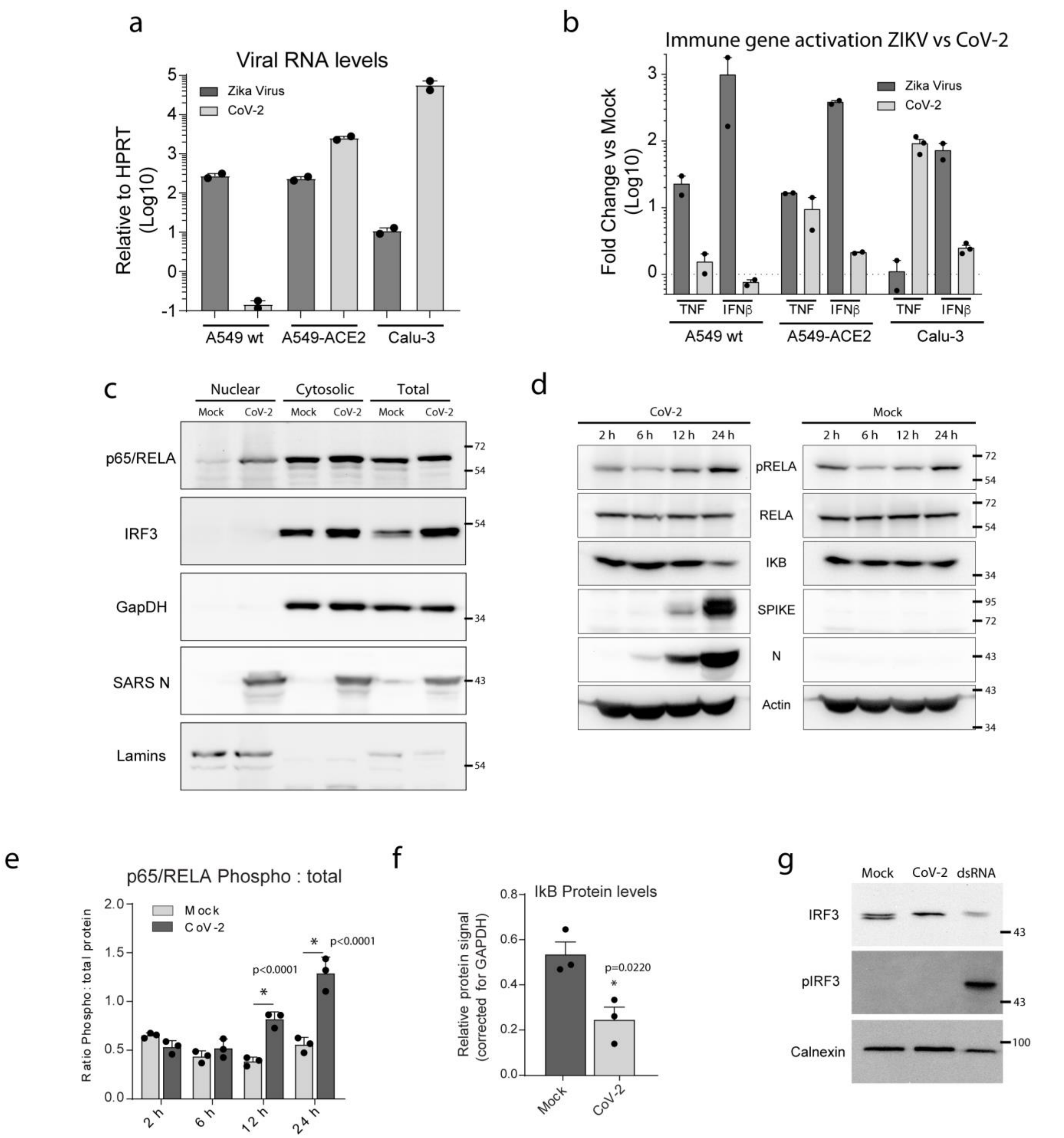

Supplementary Figure 3 | a-b, A549, A549-ACE2 or Calu-3 cells were infected with SARS-CoV-2 or Zika Virus (ZIKV) for 24 h, n=2 biological replicates. **a,** Graph shows the average viral RNA levels corrected for HPRT. **b,** Graph shows the average fold change of the indicated transcripts in infected cells compared to uninfected cells. **c,** A549-ACE-2 or Calu-3 cells were infected with SARS-CoV-2 for 16 h followed by subcellular fractionation into Nuclear, Cytosolic, and whole cell lysate (Total) fractions. Protein levels of the indicated proteins were determined by western blotting using mono-specific primary antibodies. The quantification of the blots is shown in Fig. 2 g-h. **d-f,** Cells were infected with SARS-CoV-2 for the indicated times. Cells were lysed and levels of given proteins were determined by western blot using mono-specific primary antibodies. **e,** Western blot signals for phosopho-p65/RELA (pRELA) were quantified and compared to the corresponding total p65/RELA protein levels. Graph shows the mean and

SEM for pRELA vs. total p65/RELA protein levels, n=3 biological replicates. **f,** Western blot signals for IκB were quantified and the mean ratio of signal from SARS-CoV-2 infected compared to uninfected cells is shown. n=3 biological replicates. **g,** Cells were infected with SARS-CoV-2 for 24 h or transfected with dsRNA for 6 h. Cells were lysed and the levels of given proteins were determined by western blot using mono-specific primary antibodies.

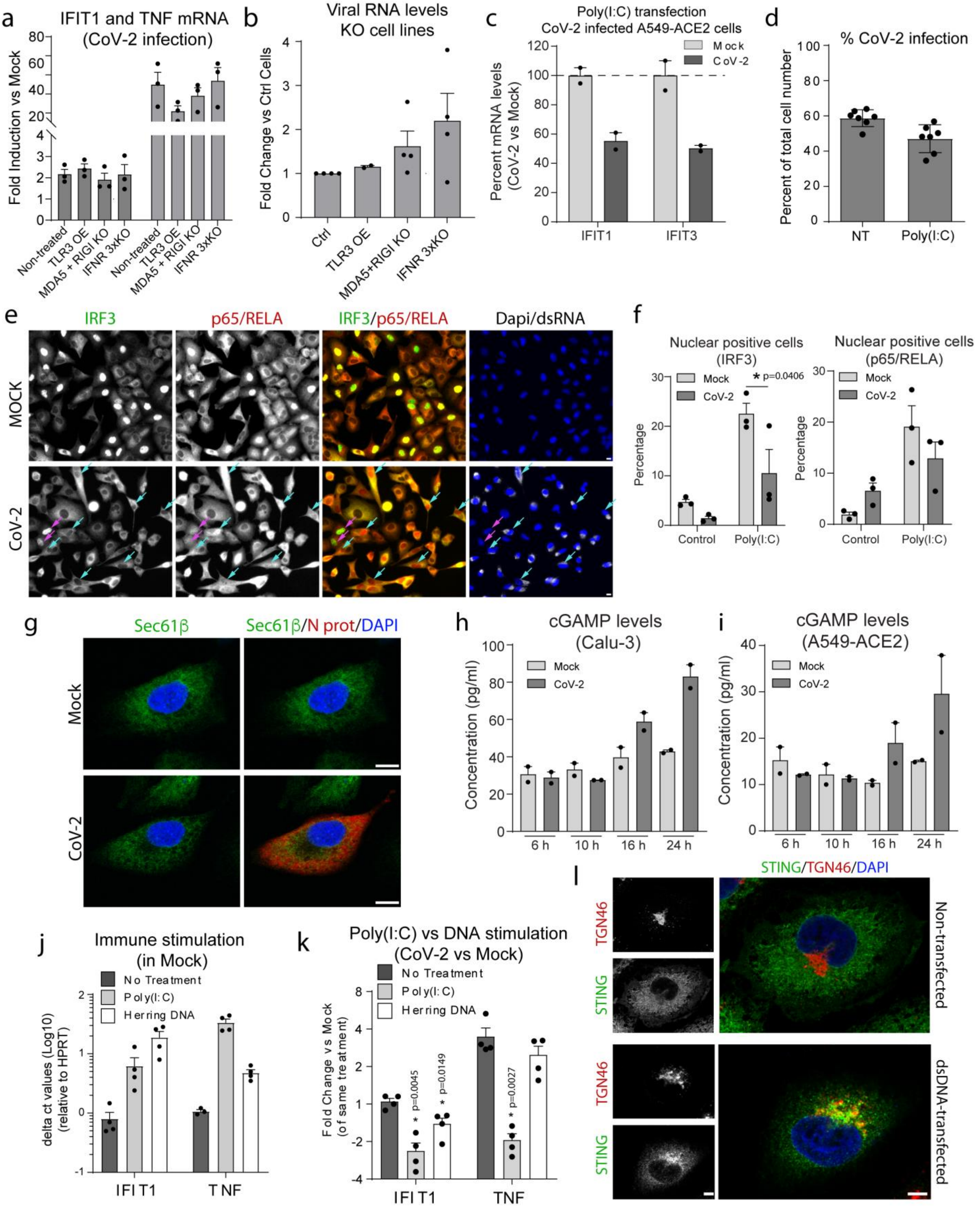

Supplementary Figure 4 | a-b, The indicated A549-ACE2 knockout cell lines were infected with SARS-CoV-2 and, 16 h after infection, total RNA was isolated and the levels of IFIT1 and TNF mRNA (a) as

well as intracellular viral RNA (b) were determined by RT-qPCR. The graphs show the means and SEMs compared to HPRT mRNA levels for 3 independent experiments. The y-axis scale is displayed in log2 increments. **c-f**, A549-ACE2 cells were infected with SARS-CoV-2 for 6 h followed by transfection with poly(I:C) and incubated for 4 h. **c,** Total RNA was isolated and the mRNA levels of IFIT1 and IFIT3 were determined by RT-qPCR. Graphs show the mean and SEM from 2 independent experiments. **d-f,** Cells were fixed and stained with antibodies specific for IRF3 (green), p65/RELA (red) and dsRNA (grey). **d,** Graph shows the average percent of infected cells for 7 fields of view collected from 3 independent experiments determined by dsRNA fluorescence signal (N>200 cells per field of view). **e,** Panel shows representative images from infected and treated cells. Magenta arrows point to cells with nuclear accumulation of both IRF3 and p65/RELA and turquoise arrows point to cells with only p65/RELA nuclear signal. Scale bars, 10 μm. **f,** Quantification of nuclear translocation of fluorescence signals from p65/RELA or IRF3 from 7 fields of view collected from 3 independent experiments conducted as in panel (e). Graphs show the mean number of cells with nuclear signal in uninfected or infected cells in either the Mock or SARS-CoV-2 treatment conditions. Infection was determined by the dsRNA signal. Quantification was done using an in-house Fiji macro. **g,** Cells were infected with SARS-CoV-2 for 16 h followed by fixation and staining with the indicated antibodies. Cells were analyzed by confocal microscopy. Scale bars 10 μm. **h-i**, Representative experiment with 2 biological replicas for figure 3c-3d. Calu-3 or A549-ACE2 cells were infected with SARS-CoV-2. Whole cell lysates from infected and uninfected cells were harvested at the indicated time points. Intracellular cGAMP levels were evaluated by ELISA and corrected for total cellular protein levels. Graphs show the average cGAMP levels (pg/mL) for uninfected and infected cells at each time-point after infection. **j-k,** Cells were infected with SARS-CoV-2 or mock-infected for 6 h, then transfected with poly(I:C) or herring DNA and 4 h thereafter, total RNA was isolated and the mRNA levels of given immune genes were determined by RT-qPCR. **j,** The graph show the mean mRNA levels of IFIT1 or TNF, corrected for HPRT, for 4 independent experiments for mock-infected cells. **k,** Analogous to panel j, but for SARS-CoV-2 infected cells. Graphs show fold change compared to mock of the same treatment for 4 independent experiments. The y-axis scale is displayed in log2 increments. **l,** A549-ACE2 cells were untreated or transfected with dsDNA. 4 h later, cells were fixed and staining with the indicated antibodies. Cells were analyzed by confocal microscopy. Scale bars 10 μm. Unless otherwise stated, n≥3 biological replicates.

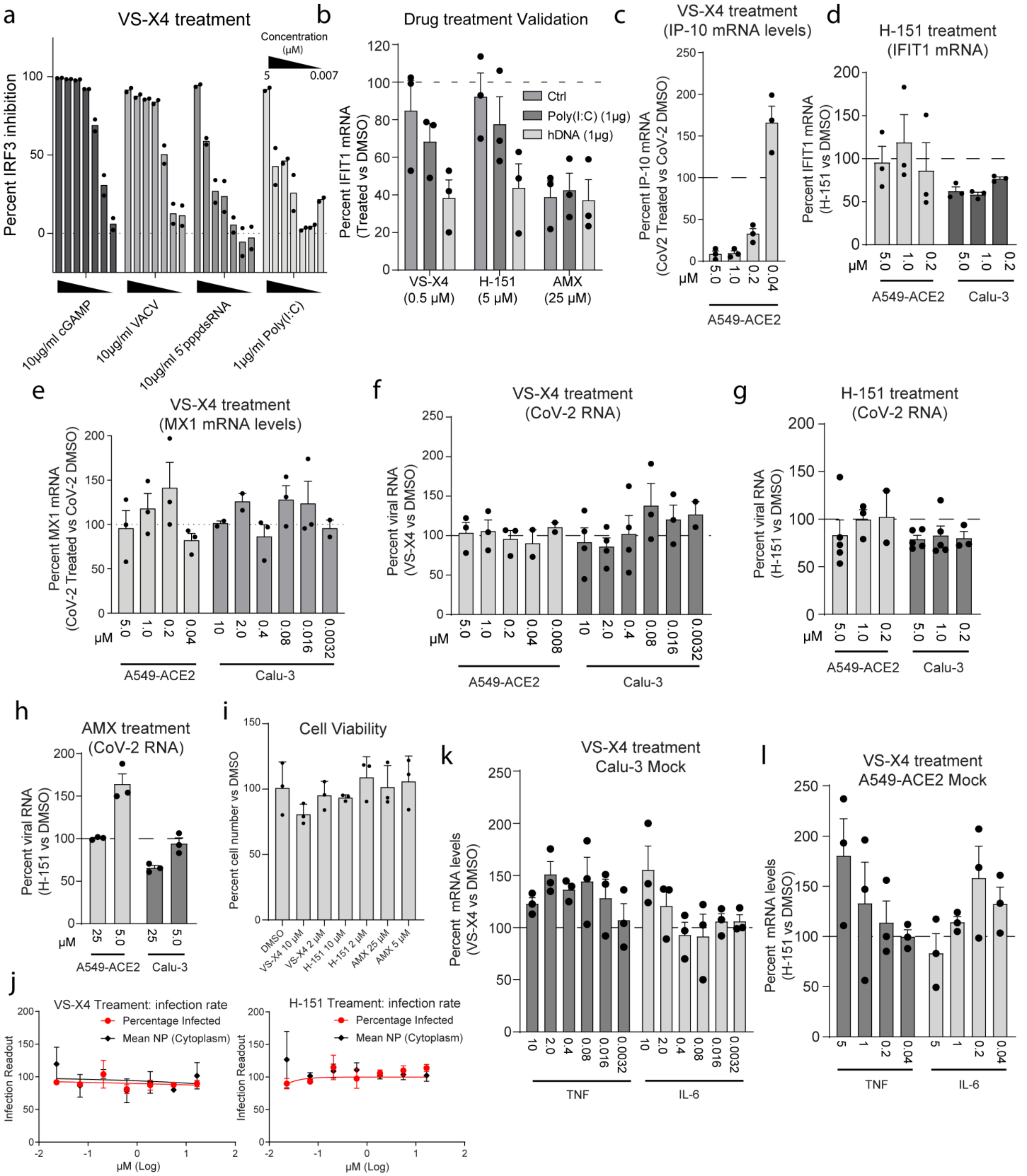

Supplementary Figure 5 | a, THP-1 dual reporter cells were treated with different concentrations (5 μM to 0.007 μM) of VS-X4 for 1 h followed by inductions with either 10 μg/ml 2',3'cGAMP, 10 μg/ml VACV-70 (dsDNA with vaccinia virus motifs), 10 μg/ml pppdsRNA, or 1 μg/ml poly(I:C). The cells were then incubated for 20 h at 37°C before measuring IRF activation by using the Quanti-Luc assay. The percent-inhibition was calculated as 100-[(luminescence of treated well/ luminescence of untreated

well)/100] X 100. IC50 values were calculated by using the Xlfit software package. n=2 biological replicates. **b,** Calu-3 cells were transfected with poly(I:C), herring DNA, or left untransfected for 10 h in the presence of the indicated drugs or DMSO. IFIT1 mRNA transcripts levels were quantified by RT-qPCR using specific primers and corrected for HPRT. Graph shows the average fold change compared to DMSO-treated samples. The y-axis scale is displayed in log2 increments. **c-l,** Cells were infected with SARS-CoV-2 and, 1 h later, cells were treated with the given drugs or DMSO only. **c-e,** Total RNA was isolated, and the IL-6 or IP-10 mRNA levels were determined by RT-qPCR. The graph shows the mean and SEM for 3 independent experiments corrected for HPRT. **f-h,** Total RNA was isolated and the viral RNA levels were determined by RT-qPCR. The graphs show the mean and SEM for 3 independent experiments corrected for HPRT. **i,** Cell viability for each drug treatment was determined by counting the mean number of cells in each well over 3 independent experiments. **j**, SARS-CoV-2 infected cells were fixed and stained with antibodies specific for the viral N protein. Graph shows the mean percent of infected cells and mean N protein (NP) fluorescence intensity for each condition. X-axis shows the log scale µM concentration of each drug starting at 50 µM. **k-l,** Total RNA was isolated from uninfected drug-treated cells and the mRNA levels of the cytokines specified on the bottom were determined by RT-qPCR. The graph shows the mean and SEM fold change vs DMSO treated cells for 3 independent experiments corrected for HPRT. Unless otherwise stated, n≥3 biological replicates.

Sup. Fig. 3 Panel c

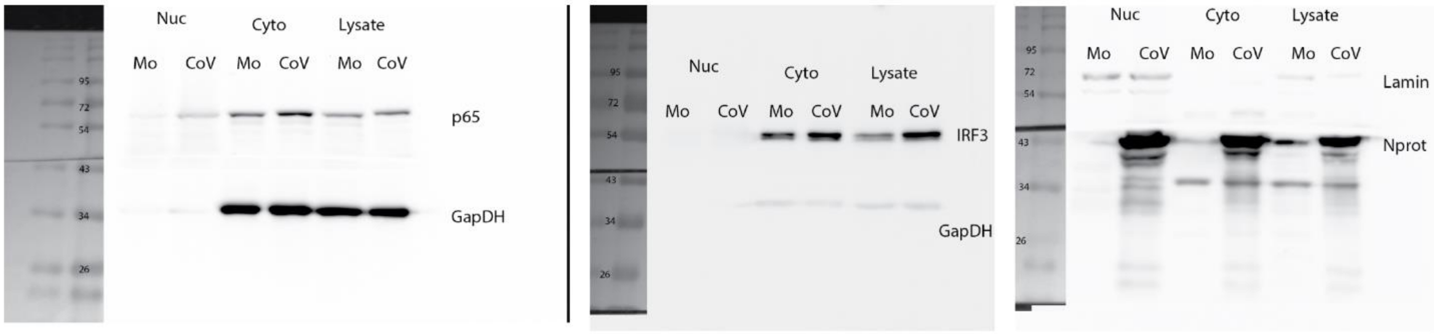

Sup. Fig. 3 Panel d

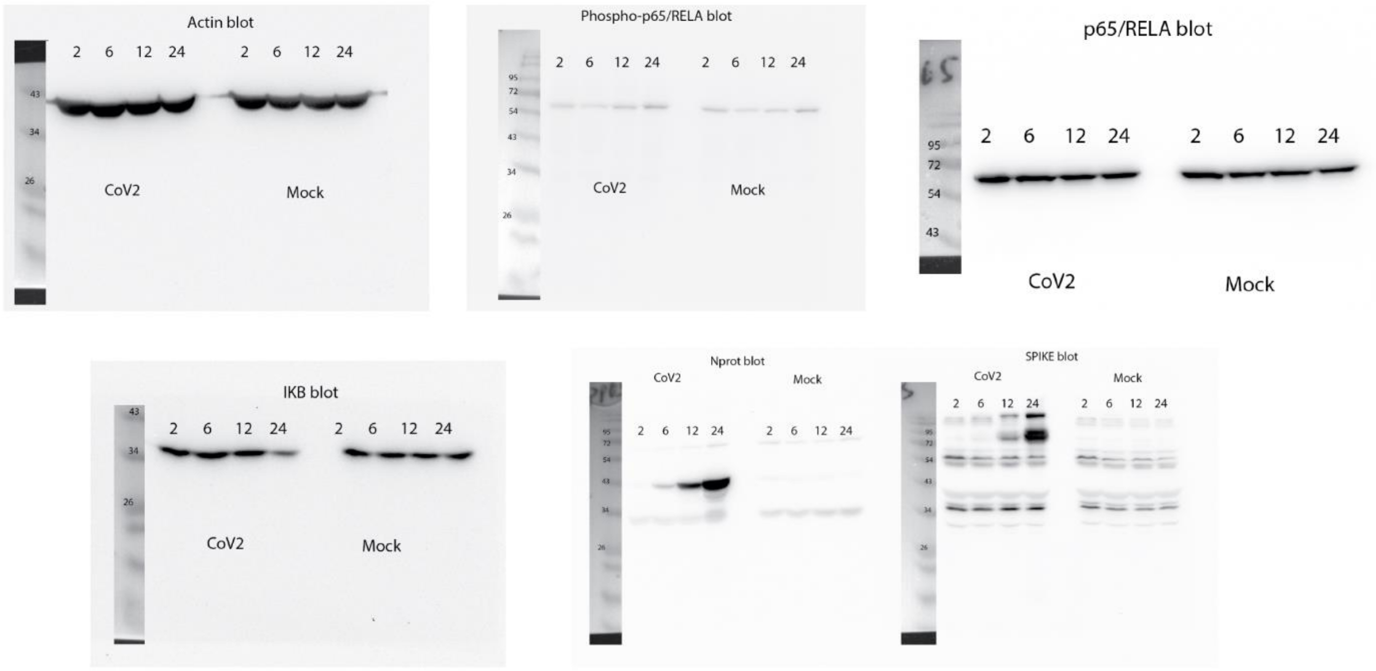

Sup. Fig. 3 Panel g

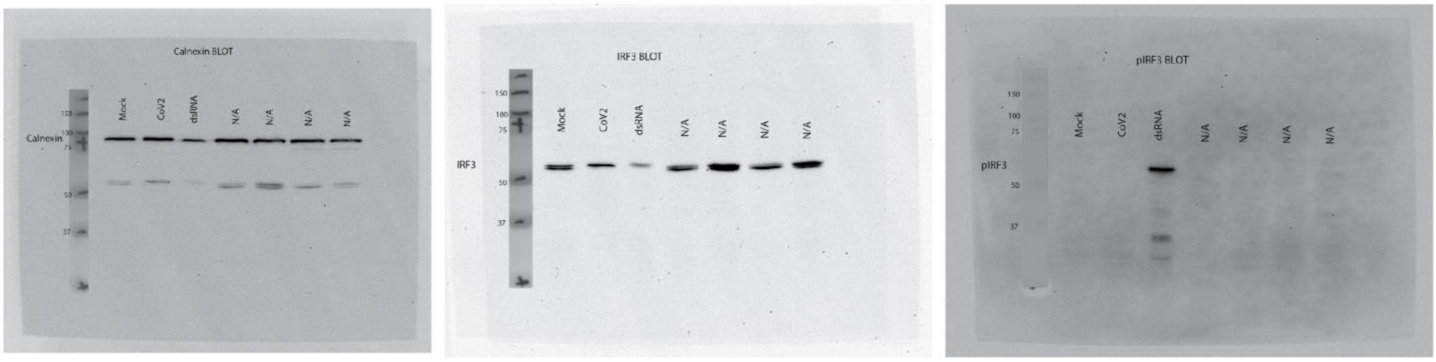

Supplementary Figure 6 | Raw western blots.
