# Supplementary material for: SARS-CoV-2 infection induces a pro-inflammatory cytokine response through cGAS-STING and NF-κB
Source: Commun Biol. 2022 Jan 12;5:45. doi: 10.1038/s42003-021-02983-5 (PMC8755718; doi:10.1038/s42003-021-02983-5)
Supplement: Supplementary file 2 — Description of Additional Supplementary Files [file 42003_2021_2983_MOESM2_ESM.pdf]

# Description of Additional Supplementary Files

**File name:** Supplementary Data 1

**Description:** Contains all the source data for graphs generated in the paper.
