# Supplementary material for: SARS-CoV-2 infection induces a pro-inflammatory cytokine response through cGAS-STING and NF-κB
Source: Commun Biol. 2022 Jan 12;5:45. doi: 10.1038/s42003-021-02983-5 (PMC8755718; doi:10.1038/s42003-021-02983-5)
Supplement: Supplementary file 4 — Reporting Summary [file 42003_2021_2983_MOESM4_ESM.pdf]

nature research

Corresponding author(s): Ralf Bartenschlager
Christopher Neufeldt

Last updated by author(s): Nov 25, 2021

Reporting Summary

Nature Research wishes to improve the reproducibility of the work that we publish. This form provides structure for consistency and transparency in reporting. For further information on Nature Research policies, see our Editorial Policies and the Editorial Policy Checklist.

Statistics

For all statistical analyses, confirm that the following items are present in the figure legend, table legend, main text, or Methods section.

n/a	Confirmed	
☐	☒	The exact sample size (*n*) for each experimental group/condition, given as a discrete number and unit of measurement
☐	☒	A statement on whether measurements were taken from distinct samples or whether the same sample was measured repeatedly
☐	☒	The statistical test(s) used AND whether they are one- or two-sided<br>*Only common tests should be described solely by name; describe more complex techniques in the Methods section.*
☐	☒	A description of all covariates tested
☐	☒	A description of any assumptions or corrections, such as tests of normality and adjustment for multiple comparisons
☐	☒	A full description of the statistical parameters including central tendency (e.g. means) or other basic estimates (e.g. regression coefficient) AND variation (e.g. standard deviation) or associated estimates of uncertainty (e.g. confidence intervals)
☒	☐	For null hypothesis testing, the test statistic (e.g. *F*, *t*, *r*) with confidence intervals, effect sizes, degrees of freedom and *P* value noted<br>*Give P values as exact values whenever suitable.*
☒	☐	For Bayesian analysis, information on the choice of priors and Markov chain Monte Carlo settings
☒	☐	For hierarchical and complex designs, identification of the appropriate level for tests and full reporting of outcomes
☐	☒	Estimates of effect sizes (e.g. Cohen's *d*, Pearson's *r*), indicating how they were calculated

Our web collection on statistics for biologists contains articles on many of the points above.

Software and code

Policy information about availability of computer code

Data collection	Confocal images were obtained with a Leica SP8 microscope using the Leica LAS X software. Wide field microscopy images were taken using a Nikon Eclipse Ti2-E inverted fluorescent microscope. All original images were taken with a bit depth of 12 bits for display images or 16 bits for images that were quantified. Images for western blot were taken using the ChemoCam 6.0 ECL system (INTAS Science Imaging, Goettingen, Germany). Chip scanning Gene Expression Microarrays were scanned using the Affymetrix GeneChip® Scanner 3000. ImageJ (National Institute of Health), CellProfiler, or CellProfiler Analyst (Broad Institute) software was used to quantify both microscopy and western blot images. qPCR results were obtained using Bio-Rad CFX Manager 3.1. Adobe Photoshop and Illustrator software packages were used to assemble images into figures.
Data analysis	Statistical analyses were performed using the GraphPad Prism 5.0 or Microsoft Excel software. Two-tailed paired Student's t-test with Bonferroni correction for multiple samples comparison or one-way ANOVA using a Dunnett post analysis were used to assess statistical significance. Data sets were considered significantly different if the P-value was less than 0.05. Cytokine arrays were processed using Bio-Plex Manager software. Microarray data analysis was done using R version 4.0.0 with the Bioconductor package, limma version 3.40.6. Gene set enrichment analysis was performed using the practical R implementation "fgsea" and the hallmark pathway gene sets. Analysis of percent infected cells was done using the EBImage platform in R-Studio.

For manuscripts utilizing custom algorithms or software that are central to the research but not yet described in published literature, software must be made available to editors and reviewers. We strongly encourage code deposition in a community repository (e.g. GitHub). See the Nature Research guidelines for submitting code & software for further information.

Data

Policy information about availability of data

All manuscripts must include a data availability statement. This statement should provide the following information, where applicable:

- Accession codes, unique identifiers, or web links for publicly available datasets
- A list of figures that have associated raw data
- A description of any restrictions on data availability

Microarray data used to support this study is deposited in Gene Expression Omnibus database repository with the accession number GSE189086, (https://www.ncbi.nlm.nih.gov/geo/query/acc.cgi?acc=GSE189086) 88. The scripts to reproduce the processed data and all figures is available online (https://github.com/boutroslab/Supp_Neufeldt_2021). Data used to produce all graphs in the manuscript can be found in Supplementary data 1. The raw western blots used to make panels in Supplementary Figure 3 can be found in Supplementary Figure 6. The remainder of the data that support the findings of this study are available from the corresponding authors upon reasonable request.

Field-specific reporting

Please select the one below that is the best fit for your research. If you are not sure, read the appropriate sections before making your selection.

☒ Life sciences ☐ Behavioural & social sciences ☐ Ecological, evolutionary & environmental sciences

For a reference copy of the document with all sections, see nature.com/documents/nr-reporting-summary-flat.pdf

Life sciences study design

All studies must disclose on these points even when the disclosure is negative.

Sample size	Experiments were performed using sample sizes based on standard protocols in the field. No statistical test was performed to predetermine sample size.
Data exclusions	No relevant data were excluded from this manuscript.
Replication	For all data shown in the paper, unless otherwise stated, experiments were replicated the indicated number of times with a minimum of 3 biological replicas. No replicates where excluded from the data.
Randomization	Sample allocation was random.
Blinding	When applicable, experiments were conducted in a blinded manner.

Reporting for specific materials, systems and methods

We require information from authors about some types of materials, experimental systems and methods used in many studies. Here, indicate whether each material, system or method listed is relevant to your study. If you are not sure if a list item applies to your research, read the appropriate section before selecting a response.

Materials & experimental systems

n/a	Involved in the study	
☐	☒	Antibodies
☐	☒	Eukaryotic cell lines
☒	☐	Palaeontology and archaeology
☒	☐	Animals and other organisms
☐	☒	Human research participants
☒	☐	Clinical data
☒	☐	Dual use research of concern

Methods

n/a	Involved in the study	
☒	☐	ChIP-seq
☒	☐	Flow cytometry
☒	☐	MRI-based neuroimaging

Antibodies

Antibodies used	Primary antibodies: Mouse anti-dsRNA J2 (Scicons: 10010500, IF-1:1000); Mouse anti-SARS-CoV-2 N protein (Sino Biological: 40143-MM05, IF - 1:1000; WB - 1:1000); Rabbit anti-SARS-CoV-2 Spike protein (Abcam: ab252690, WB- 1:1000); Rabbit anti-IRF3 (Cell Signaling Technology: 11904S, IF - 1:400); Mouse anti-P65/RELA (Santa Cruz: sc-8008, IF - 1:100); Rabbit anti-cGAS (Atlas Antibodies: HPA031700, IF – 1:100); Rabbit anti-STING (Atlas Antibodies: HPA038534, IF – 1:100); Mouse anti-dsDNA (Abcam: ab27156, IF – 1:2000); Rabbit anti-p65/RELA (Cell Signaling: L8F6, WB – 1:1000); Rabbit anti-phospho-p65/RELA (Cell Signaling: 3033, WB - 1:1000); Rabbit anti-IkB (Cell Signaling: 9242s, WB – 1:1000); Sheep and-TGN46 (Biorad:AHP500G, IF-1:200); Mouse anti-Actin (Sigma Aldrich: A5441, WB-1:5000); Rabbit anti-HA (Thermo Fisher PA1-985 IF-1:500); Rabbit anti-pIRF3 (Cell Signaling: 4947, WB – 1:1000); Mouse anti-PDI (Thermo Fisher: MA3-019, IF-1:200); Mouse anti-IL-6 (R&D systems: MAB2061-100, Neutralization 1:1000); Goat anti-TNF

	(R&D systems: AF-410-NA, Neutralization 1:5000); rabbit anti-IL-6 (Thermo: P620, WB 1:500); Rabbit anti-TNF (Thermo: AMC3012, WB 1:500); Mouse anti-LaminA/C (SantaCruz: sc-7292, WB 1:1000).<br>Secondary antibodies: Goat anti–rabbit IgG-HRP (Sigma Aldrich A6154, 1:2000), Goat anti–mouse IgG-HRP (Sigma Aldrich A4416, 1:5000), Alexa Fluor 488 donkey anti-rabbit IgG (Thermofisher A-21206), Alexa Fluor 488 donkey anti-mouse IgG (Thermofisher A-21202), Alexa Fluor 488 donkey anti-mouse IgG2a (Thermofisher A-21131), Alexa Fluor 568 donkey anti-rabbit IgG (Thermofisher A-10042), Alexa Fluor 568 donkey anti-mouse IgG (Thermofisher A-10037), Alexa Fluor 568 donkey anti-mouse IgG1 (Thermofisher A-21124), Alexa Fluor 647 donkey anti-rabbit IgG (Thermofisher A -31573), Alexa Fluor 647 donkey anti-mouse IgG (Thermofisher A-31571), (ALL Alexa fluor secondaries used at 1:1000)
Validation	All antibodies were validated by immunofluorecence microscopy or western blot using sub-cellular localization, protein size, or functional readouts to verify specificity.

Eukaryotic cell lines

Policy information about cell lines

Cell line source(s)	VeroE6 were obtained from progen<br>A549 cells were obtained from ATCC<br>A549-ACE2 cells: doi:10.1016/j.chom.2020.11.003 (2020)<br>Calu-3: doi:10.1016/j.chom.2020.11.003 (2020)
Authentication	The cell lines used were not authenticated.
Mycoplasma contamination	All cell lines are regularly tested for mycoplasma contamination and were negative.
Commonly misidentified lines (See ICLAC register)	A549 cells were used in this study as a lung cell model for SARS-CoV-2 infection.

Human research participants

Policy information about studies involving human research participants

Population characteristics	Participants were determined by infection with SARS-CoV-2 and presentation with severe COVID-19 symptoms.
Recruitment	Patients were recruited consecutively and prospectively. As inclusion criterion ventilated COVID-19 patients were selected.
Ethics oversight	Ethics Committee of the Medical Faculty Heidelberg (ethics approval number S-148/2020)

Note that full information on the approval of the study protocol must also be provided in the manuscript.
